# Enhanced Band‐Crystal Engineering Drives Superior Power Generation in GeTe

**DOI:** 10.1002/advs.202506612

**Published:** 2025-05-22

**Authors:** Xiaobo Tan, Qian Deng, Jianglong Zhu, Ruiheng Li, Xuri Rao, Fan Feng, Shuang Lyu, Pengfei Nan, Yue Chen, Binghui Ge, Ran Ang

**Affiliations:** ^1^ Key Laboratory of Radiation Physics and Technology Ministry of Education Institute of Nuclear Science and Technology Sichuan University Chengdu 610064 China; ^2^ Department of Mechanical Engineering The University of Hong Kong Pokfulam Road Hong Kong SAR 999077 China; ^3^ Information Materials and Intelligent Sensing Laboratory of Anhui Province Leibniz International Joint Research Center of Materials Sciences of Anhui Province Institutes of Physical Science and Information Technology Anhui University Hefei 230601 China; ^4^ College of Physics Sichuan University Chengdu 610064 China; ^5^ Institute of New Energy and Low‐Carbon Technology Sichuan University Chengdu 610065 China

**Keywords:** band engineering, conversion efficiency, crystal structure, GeTe, thermoelectrics

## Abstract

Optimizing both electrical and thermal performance in thermoelectric (TE) materials is challenging due to the inherent coupling between carrier and phonon transport. To address this, targeted modulation of band structure and crystal lattice is achieved in the optimized Ge_0.885_Zr_0.02_Pb_0.08_Te_0.985_(Cu_2_Te)_0.015_ sample. Zr/Pb incorporation optimizes the band structure and significantly enhances the Seebeck coefficient, while Pb‐substituted Ge sites occupy a more symmetric geometric center, reducing Ge vacancies, increasing crystal symmetry, and facilitating delocalized carrier transport. This leads to optimized carrier‐weighted mobility (*µ*_w_) ≈210 cm^2^ V^−1^ S^−1^ (average power factor ≈30.3 µW cm^−1^ K^−2^). Moreover, the alteration of this geometric center enhances phonon anharmonicity, and multi‐scale defect structures induced by multi‐element doping provide abundant phonon scattering sources. Consequently, the sample exhibits significantly improved *µ*_w_/*κ*_L_ values over pristine GeTe across the entire temperature range, with an improvement of ≈238% at 650 K. A peak *zT* of ≈2.2 at 650 K translates to a maximum heat‐to‐electricity conversion efficiency of up to 8.5% for a 7‐pair device at Δ*T* = 366 K. This work further reveals the potential of synergistic band and crystal control engineering in decoupling carrier and phonon transport in GeTe‐based materials, paving the way for broader applications of GeTe‐based TE devices.

## Graphical abstract

By precisely tuning the band structure and crystal structure in GeTe‐based thermoelectric (TE) materials, this study achieves comprehensive control over electron transport and phonon scattering. Structural optimization significantly enhances carrier‐weighted mobility (*µ*_w_) while effectively suppressing lattice thermal conductivity (*κ*_L_), resulting in a TE performance with a *zT* of ≈2.2 and a device efficiency of up to ≈8.5%. This work presents a crucial strategy for advancing TE conversion technologies.

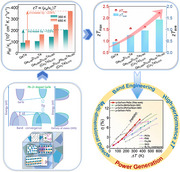

## 1 Introduction

Energy waste remains an irreversible global challenge that is attracting increasing attention.^[^ ^1^, ^2^ ^]^ Thermoelectric (TE) technology offers promising solutions for converting dispersed waste heat into electricity, thereby enhancing overall energy conversion efficiency.^[^ ^3^, ^4^, ^5^, ^6^ ^]^ The effectiveness of TE materials is measured by the dimensionless figure of merit (*zT*), defined as *zT* = *S* ^2^ *σT*/*κ*_total_, where *S* denotes the Seebeck coefficient, *σ* refers to the electrical conductivity, *T* represents the absolute temperature in kelvin, and *κ*_total_ stands for the total thermal conductivity, encompassing both electronic (*κ*_e_) and lattice (*κ*_L_) contributions.^[^ ^7^, ^8^, ^9^ ^]^ To achieve high TE performance, it is essential to optimize both the power factor (*PF*) for electrical transport and the *κ*_L_ for thermal management.^[^ ^10^, ^11^ ^]^ A higher ratio of carrier‐weight mobility (*µ*_w_) to *κ*_L_—denoted as *µ*_w_/*κ*_L_—is often a reliable indicator of superior TE material properties.^[^ ^12^, ^13^, ^14^, ^15^ ^]^ However, due to the strong coupling of these parameters, improving one often comes at the expense of another, making it challenging to achieve optimal TE performance.^[^ ^16^, ^17^, ^18^, ^19^, ^20^, ^21^ ^]^ Additionally, for practical applications, TE materials need to maintain high efficiency across a wide temperature range to maximize overall energy conversion efficiency (*η*). Therefore, optimizing the average *zT_ave_* over the relevant temperature range is a crucial strategy for enhancing material optimization.

Among the commonly studied high‐performance TE materials, main group chalcogenides, such as the wide‐bandgap semiconductor SnS (*E*_g_ ≈1.16 eV),^[^ ^22^ ^]^ are capable of mitigating the detrimental effects of high‐temperature intrinsic excitation on TE performance. However, their wide bandgap leads to low intrinsic carrier density, which inherently limits their electrical performance. On the other hand, narrow‐bandgap semiconductors like (Bi, Sb)_2_Te_3_ (*E*_g_ ≈0.13 eV),^[^ ^23^ ^]^ PbTe (*E*_g_ ≈0.28 eV),^[^ ^24^ ^]^ PbSe (*E*_g_ ≈0.27 eV),^[^ ^25^ ^]^ and SnTe (*E*_g_ ≈0.18 eV)^[^ ^26^ ^]^ exhibit electronic properties that can be more easily tuned to enhance electrical performance. However, these materials are often limited by severe bipolar conduction effects at elevated temperatures, which degrade their *zT* values. In contrast, GeTe (*E*_g_ ≈0.585 eV) emerges as a distinctive narrow‐bandgap semiconductor due to its unique ability to undergo band structure redistribution driven by structural symmetry changes. This band restructuring enhances charge carrier transport, making GeTe a promising candidate for high‐performance TE applications. The favorable electronic band structure of GeTe not only facilitates efficient charge transport but also has historically been a key factor in selecting high‐efficiency TE materials.^[^ ^27^, ^28^ ^]^

It is widely accepted that the microscopic transport characteristics of charge carriers are pivotal in determining the *PF* of TE materials, significantly impacting the output power of TE devices.^[^ ^29^, ^30^ ^]^ GeTe, in particular, exhibits remarkable potential for optimizing electrical performance due to its ability to undergo a continuous phase transition from cubic‐GeTe to rhombohedral‐GeTe. This structural phase transition, occurring along the [111] crystallographic direction near ≈720 K, induces a polar crystal structure transformation that modifies electron localization, resulting in substantial alterations to the band structure.^[^ ^13^, ^31^, ^32^ ^]^ By enhancing crystal symmetry to mitigate electron localization and employing band engineering strategies to increase effective band degeneracy,^[^ ^33^, ^34^, ^35^, ^36^ ^]^ the intrinsic trade‐off between Hall carrier mobility (*µ*_H_) and effective mass (*m* ^*^) can be effectively decoupled. This decoupling leads to an improvement in carrier‐weighted mobility (*µ*_w_ = *µ*_H_(*m* ^*^/*m*_e_)^3/2^), thereby significantly enhancing electrical performance across a wide temperature range. Such advancements are crucial for achieving higher power outputs in TE devices.^[^ ^30^, ^37^ ^]^ However, pristine GeTe typically suffers from unintentional doping, resulting in an excessively high hole carrier concentration (*n*_H_) due to low defect formation energies. This leads to the evaporation of Ge atoms from the lattice, yielding a substantial *n*_H_ of ≈1 × 10^21 ^cm^−3^.^[^ ^38^, ^39^, ^40^ ^]^ The elevated *n*_H_ intensifies carrier scattering, which adversely affects *µ*_H_ and contributes to a higher 𝜅_e_.^[^ ^41^, ^42^ ^]^ Addressing these challenges is essential for further optimizing the TE performance of GeTe‐based materials.

Therefore, heterovalent doping and vacancy manipulation are critical strategies for optimizing *n*_H_, as well as fine‐tuning defects and microstructures to minimize *κ*_L_.^[^ ^39^, ^41^, ^43^ ^]^ The narrow bandgap of GeTe, however, limits its TE performance to specific temperature ranges, necessitating doping or alloying to widen the bandgap and improve average TE performance.^[^ ^44^ ^]^ Yet, these modifications often introduce undesirable effects, such as increased point defects or stacking faults, which can further degrade *µ*_H_. To address this challenge, substituting dopants for atomic sites within the GeTe matrix can create local atomic disorder or form ordered Ge‐vacancy layers (i.e., van der Waals gap), thereby enhancing phonon scattering without impairing carrier transport. This dual approach effectively reduces *κ*_L_ while preserving high *µ*_H_.^[^ ^36^, ^45^ ^]^ Moreover, selecting dopants based on similar atomic electronegativity proves to be an effective strategy for screening materials with high *µ*_H_.^[^ ^46^, ^47^ ^]^ A large electronegativity difference (*Δχ*) can negatively impact *µ*_H_ due to ionic bonding and increased polar phonon scattering.^[^ ^48^ ^]^ In contrast, a smaller *Δχ*—for example, *Δχ* = 0.3 for Ge–Te and 0.2 for Pb–Te and Cu–Te—can mitigate these issues, thus optimizing the electrical properties of the TE material.

**Figure 1:**
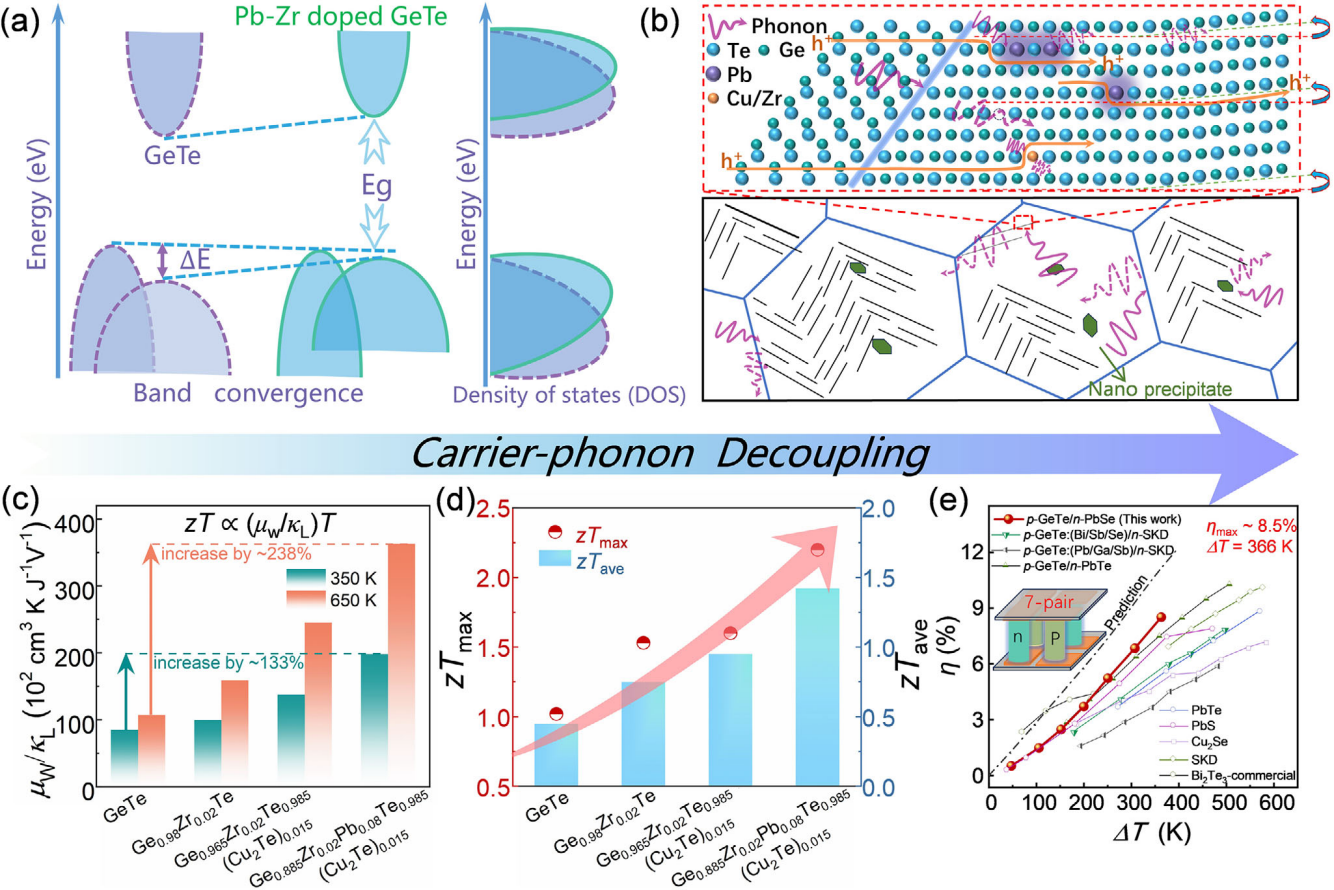
Strategies for enhancing the thermoelectric efficiency of Pb‐doped GeTe‐based materials. a) Schematic representation of the dynamic evolution in the band structure along with the corresponding density of states (DOS). b) Illustration of the effects of a multiscale hierarchical structure on phonon scattering and transport within the Ge_0.885_Zr_0.02_Pb_0.08_Te_0.985_(Cu_2_Te)_0.015_ sample. c) *µ*_w_/*κ*_L_ values at 350 and 650 K, where *µ*_w_ is calculated using the method proposed by Snyder et al.^[^ ^12^ ^]^ d) Comparative analysis of the temperature‐dependent maximum *zT*_max_ and the corresponding average *zT*_ave_ for the samples with GeTe, Ge_0.98_Zr_0.02_Te, Ge_0.965_Zr_0.02_Te_0.985_(Cu_2_Te)_0.015_, and Ge_0.885_Zr_0.02_Pb_0.08_Te_0.985_(Cu_2_Te)_0.015_. e) Measured conversion efficiency *η* of the *p*‐GeTe/*n*‐PbSe thermoelectric device as a function of temperature difference (Δ*T*), with benchmark data for GeTe, PbTe, Cu_2_Se, skutterudites (SKD), and Bi_2_Te_3_ devices included for comparison.^[^ ^29^, ^49^, ^50^, ^51^, ^52^, ^53^, ^54^, ^55^ ^]^

In this study, we achieve effective electron‐phonon decoupling in GeTe by manipulating its electronic structure and lattice dynamics, as illustrated in **Figure** **1**. Density functional theory (DFT) calculations and single parabolic band (SPB) model fitting confirm that Pb and Zr co‐doping promotes band convergence in GeTe, thereby increasing both the density of states (DOS) *m* ^*^ and the bandgap (Figure 1a). Additionally, Pb substitution at Ge sites optimizes *n*_H_, shifts the geometric center, and reduces the elemental electronegativity difference. This adjustment facilitates delocalized electron transport, resulting in high *µ*_w_ and excellent electrical performance. Furthermore, increased lattice anharmonicity leads to reduced sound velocity and enhanced phonon scattering. Theoretical models reveal the influence of defect structures on phonon behavior: Pb doping introduces local heterogeneity, including point defects, Cu‐rich nanoprecipitates, vacancy layers, and low‐angle grain boundaries, which collectively scatter high‐ and intermediate‐frequency phonons (Figure 1b). As a result, the optimized Ge_0.885_Zr_0.02_Pb_0.08_Te_0.985_(Cu_2_Te)_0.015_ sample exhibits improvements in the *µ*_w_/*κ*_L_ ratio by ≈133% at 350 K and ≈238% at 650 K compared to pristine GeTe (Figure 1c), continuously enhancing its TE performance (Figure 1d). The peak *zT* achieved is ≈2.2 at 650 K, with an average *zT_ave_* of ≈1.4 from 300 to 800 K. Moreover, the material demonstrates robust mechanical properties, evidenced by a Vickers microhardness of ≈200 *H*_v_, and achieves a maximum device conversion efficiency (*η*_max_) of ≈8.5% under a temperature difference (*ΔT*) of 366 K. This surpasses most reported GeTe‐based and other TE devices (Figure 1e).^[^ ^29^, ^49^, ^50^, ^51^, ^52^, ^53^, ^54^, ^55^ ^]^ This work opens new avenues for enhancing the overall performance of GeTe‐based TE devices.

## 2 Results and Discussion

To optimize the TE performance of GeTe, we implemented a strategy combining Pb doping with 1.5% Cu_2_Te alloying, resulting in the successful synthesis of a series of high‐quality polycrystalline Zr‐doped GeTe and Ge_0.965‐_ *_x_* Zr_0.02_Pb*_x_* Te_0.985_(Cu_2_Te)_0.015_ (*x =* 0–0.12) samples. The powder X‐ray diffraction (XRD) patterns confirmed a good match with the rhombohedral GeTe structure (space group *R3m*, PDF # 47–1079) (Figure S1, Supporting Information). Due to the inherently high concentration of cation vacancies in GeTe, Ge precipitates were detected in the diffraction peaks of all samples.^[^ ^32^, ^56^, ^57^ ^]^ Rietveld refinement analysis revealed that the lattice parameters (a) for the Zr‐doped GeTe samples exhibited a contraction (from 6.002 to 5.978 Å), while the Ge_0.965‐_ *_x_* Zr_0.02_Pb*_x_* Te_0.985_(Cu_2_Te)_0.015_ (*x =* 0–0.12) samples showed an expansion (from 5.989 to 6.028 Å) with increasing doping content (Figure S2a,b, Supporting Information). This variation in lattice parameters can be attributed to the differences in ionic radii: Zr^4+^ (0.86 Å), Cu^1+^ (0.91 Å), and Pb^2+^ (1.2 Å), relative to Ge^2+^ (0.87 Å).^[^ ^58^ ^]^ The primary double peaks (024) and (220) of GeTe, Ge_0.98_Zr_0.02_Te, Ge_0.965_Zr_0.02_Te_0.985_(Cu_2_Te)_0.015_, and Ge_0.885_Zr_0.02_Pb_0.08_Te_0.985_(Cu_2_Te)_0.015_ gradually merged and exhibited a characteristic shift toward the cubic‐GeTe phase (Figure S1b, Supporting Information). This trend indicates effective alloying in the samples and enhances the crystal symmetry of GeTe.^[^ ^27^, ^59^, ^60^ ^]^ Furthermore, the observed increase in the interaxial angle (*a*) supports the bimodal changes in XRD patterns (Figure S2c, Supporting Information). Overall, achieving a highly symmetric cubic structure along with a significantly distorted lattice in GeTe thermoelectrics is beneficial, as it leads to a higher *PF* and minimized *κ*_L_, respectively.^[^ ^27^, ^36^, ^59^ ^]^

**Figure 2:**
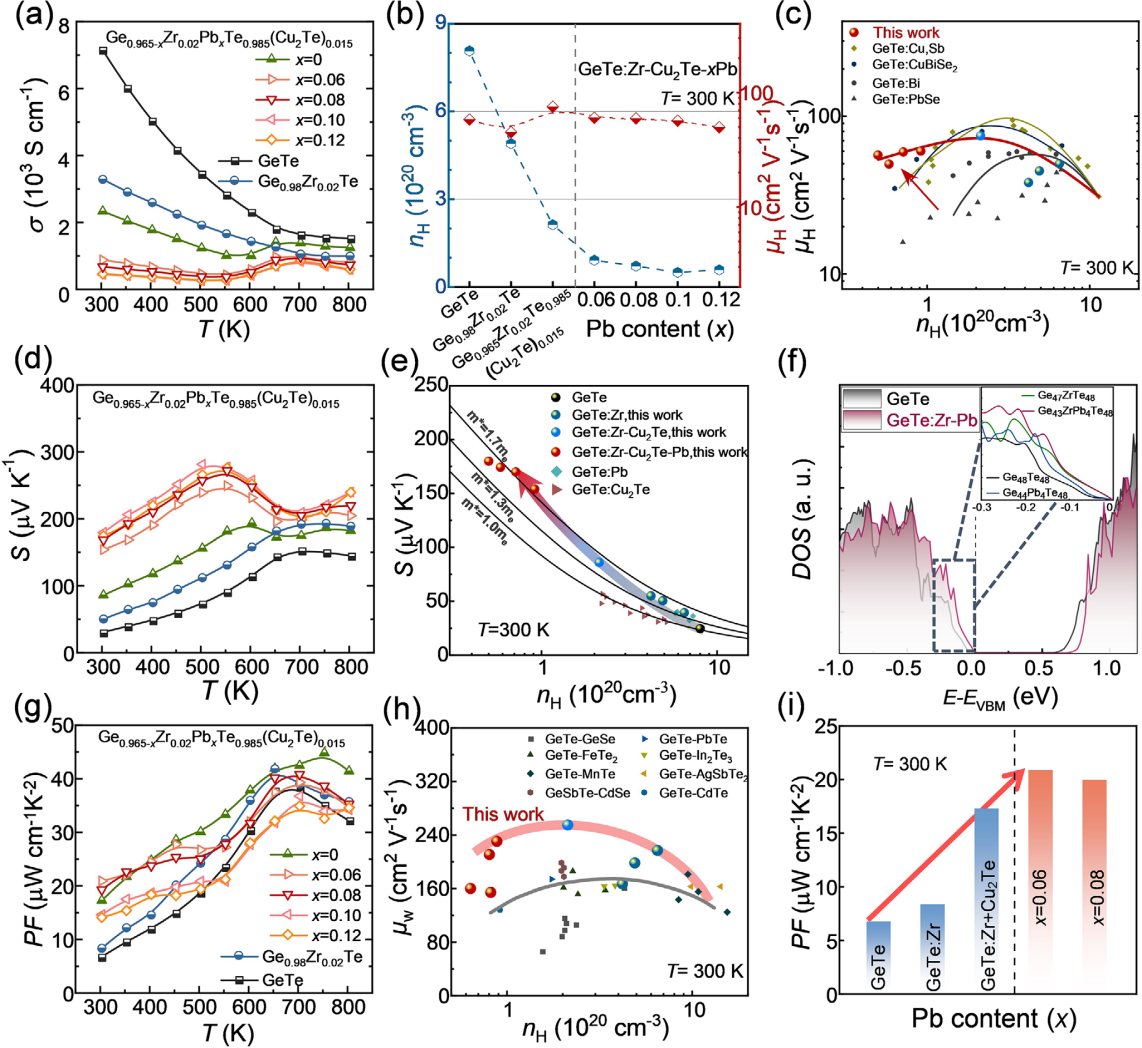
Electrical transport properties of Ge_0.965‐_ *_x_* Zr_0.02_Pb*_x_* Te_0.985_(Cu_2_Te)_0.015_ samples. a) Seebeck coefficient *S*. b) Carrier concentration *n*_H_ and carrier mobility *µ*_H_ as functions of increasing Pb content *x* content. c) Comparison of *µ*_H_, with values reported in the literature.^[^ ^39^, ^42^, ^46^, ^56^ ^]^ d) electrical conductivity *σ*. e) *S* as a function of *n*_H_, with data from the literature; solid lines represent theoretical Pisarenko curves based on the single parabolic band (SPB) model.^[^ ^13^, ^61^ ^]^ f) Density of states (DOS) for Ge_48_Te_48_, Ge_44_Pb_4_Te_48_, Ge_47_ZrTe_48_, and Ge_43_Pb_4_ZrTe_48_ compositions_._ g) Power factor *PF*. h) Weight mobility *µ*_w_ compared with literature data.^[^ ^33^, ^34^, ^62^, ^63^, ^64^, ^65^, ^66^, ^67^ ^]^ i) *PF* at room temperature.

The *σ* and *S* as functions of temperature exhibit typical behavior for *p*‐type GeTe‐based materials. As shown in **Figure** **2**a and Figures S3–S5 (Supporting Information), *σ* sharply decreases with increasing Zr donor content, while further alloying with 1.5% Cu_2_Te and Pb doping results in a marked reduction in *σ*. Hall measurements were performed to determine the *n*_H_ and *µ*_H_ of the samples, facilitating an in‐depth analysis of the electrical behavior of doped GeTe. As illustrated in Figure 2b and Figure S6 (Supporting Information), Zr^4+^ serves as an electron donor by substituting Ge^2+^ ions, effectively lowering *n*_H_ from ≈8 × 10^20^ cm^−3^ in pristine GeTe to ≈4.2 × 10^20^ cm^−3^ in the Ge_0.98_Zr_0.02_Te matrix samples at room temperature. The strong optimization effect of Cu_2_Te and Pb doping on intrinsic Ge vacancies further reduces *n*_H_ to outstanding levels of ≈2.13 × 10^20^ cm^−3^ and ≈5 × 10^19^ cm^−3^, respectively. Notably, as *n*_H_ is gradually optimized to the optimal level, the overall *µ*_H_ at room temperature remains stable and higher than that of typical GeTe‐based materials, as shown in Figure 2c. This stability is primarily attributed to the smaller *Δχ* of Pb and Cu compared to Ge–Te, as well as the enhanced lattice symmetry. Together, these factors effectively reduce carrier scattering and significantly improve the transport process.^[^ ^39^, ^42^, ^46^, ^56^ ^]^ This improvement is further corroborated by subsequent transmission electron microscopy (TEM) images and supporting literature, which emphasize the substantial enhancement of electrical transport properties.^[^ ^56^, ^61^ ^]^

**Figure 3:**
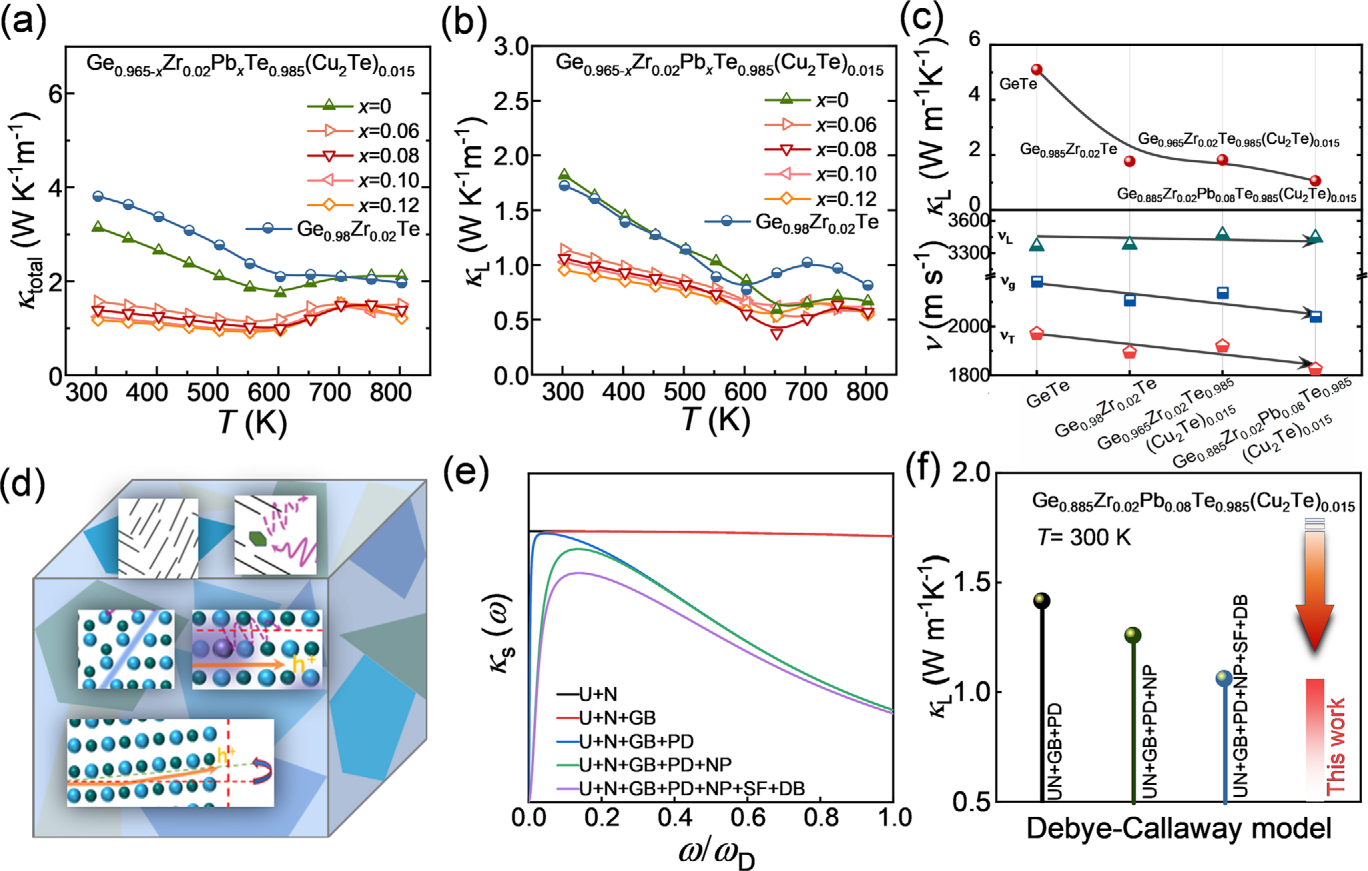
Thermal properties of Ge_0.965‐_ *_x_* Zr_0.02_Pb*_x_* Te_0.985_(Cu_2_Te)_0.015_ samples. Temperature‐dependent a) total thermal conductivity (*κ*_total_) and b) lattice thermal conductivity (*κ*_L_). c) Measured sound velocities at room temperature, including average sound velocity (*v*_g_), transverse velocity (*v*_T_), and longitudinal velocity (*v*_L_), alongside corresponding *κ*_L_ values. d) Schematic representation of the multi‐scale phonon scattering mechanisms. e) Fitted spectral lattice thermal conductivity (*κ*_s_). f) Calculated *κ*_L_ using the Debye–Callaway model for the Ge_0.885_Zr_0.02_Pb_0.08_Te_0.985_(Cu_2_Te)_0.015_ sample at 300 K, illustrating contributions from different phonon scattering mechanisms.

As expected and shown in Figure 3d, the temperature dependence of *S* exhibits a dramatic increase due to its coupling relationship with *σ*, rising from ≈50.4 µV K^−1^ in Ge_0.98_Zr_0.02_Te to ≈169 µV K^−1^ in Ge_0.885_Zr_0.02_Pb_0.08_Te_0.985_(Cu_2_Te)_0.015_ at 300 K. Notably, the peak *S* of the 1.5% Cu_2_Te alloyed sample shifted slightly to lower temperatures. While *S* decreased at 550 K due to high‐temperature intrinsic excitation, the Ge_0.965‐_ *_x_* Zr_0.02_Pb*_x_* Te_0.985_(Cu_2_Te)_0.015_ samples exhibited enhanced *S* across the entire temperature range, with a significant increase to ≈281 µV K^−1^ at 550 K. Furthermore, we analyzed the relationship between *m* ^*^ and *S* versus *n*_H_ using the SPB model, comparing our results with those from the literature, as shown in Figure 2e. Overall, *m* ^*^ was significantly enhanced in all samples, with the largest *m* ^*^ achieved in Ge_0.98_Zr_0.02_Te. Cu_2_Te alloying primarily optimized *n*_H_, while Pb doping in GeTe led to a slight increase in *m* ^*^ alongside a significant reduction in *n*_H_. Compared with literature data,^[^ ^13^, ^61^ ^]^ the synergistic effect of Cu_2_Te alloying and Pb doping resulted in improvements even at lower *n*_H_ ranges, ultimately increasing *m* ^*^ to 1.7 *m*_e_ at 300 K. Additionally, DFT calculations were performed to assess the variations in the DOS and band structures in the supercells of Ge_48_Te_48_, Ge_44_Pb_4_Te_48_, Ge_47_ZrTe_48_, and Ge_43_Pb_4_ZrTe_48_. The DOS calculations, shown in Figure 2f and Figure S7 (Supporting Information), demonstrate a significant increase near the band edges compared to Ge_48_Te_48_, with the Fermi level approaching the valence band. This validates the increased energy band mass under theoretical models. Combined with the XRD results, the change in the interaxial angle from 88.2° to 88.7° theoretically confirms that the increase in crystal symmetry promotes band degeneracy, exhibiting a distorted DOS near the valence band maximum (VBM). Furthermore, compared to pristine GeTe (*E*_g_ = 0.585 eV), the bandgap increases after Pb doping (*E*_g_ = 0.618 eV), and further expands to 0.688 eV with the synergistic effect of Zr and Pb co‐doping (Figure S7b, Supporting Information). This confirms that significant modulation of band structure degeneracy and bandgap expansion in Pb‐doped Ge_0.965‐_ *_x_* Zr_0.02_Pb*_x_* Te_0.985_(Cu_2_Te)_0.015_ leads to improved *S*. Consequently, the intrinsic Ge_0.98_Zr_0.02_Te exhibits a *PF* of ≈8.4 µW cm^−1^ K^−2^ at 300 K, while Ge_0.885_Zr_0.02_Pb_0.08_Te_0.985_(Cu_2_Te)_0.015_ achieves an optimal *PF* ≈19.3 µW cm^−1^ K^−2^ at 300 K and ≈40.8 µW cm^−1^ K^−2^ at 700 K (Figure 2g).

The *µ*_w_ is a crucial parameter that comprehensively evaluates effective *m* ^*^ and *µ*_H_, providing valuable insights for optimizing carrier transport characteristics as the band structure evolves.^[^ ^12^, ^68^, ^69^ ^]^ As shown in Figure 2h, the introduction of Cu_2_Te and Pb significantly optimized *µ*_w_ compared to the Ge_0.98_Zr_0.02_Te matrix samples. When compared to other optimization strategies in GeTe at room temperature, these samples exhibit superior performance in terms of carrier density.^[^ ^33^, ^34^, ^62^, ^63^, ^64^, ^65^, ^66^, ^67^ ^]^ Consequently, the increase in room‐temperature *PF* of the Pb‐doped Ge_0.885_Zr_0.02_Pb_0.08_Te_0.985_(Cu_2_Te)_0.015_ samples corresponds well with the significantly enhanced *µ*_w_ (Figure 2i). Ge_0.885_Zr_0.02_Pb_0.08_Te_0.985_(Cu_2_Te)_0.015_ maintains an average *PF* of ≈30.3 µW cm^−1^ K^−2^ from 300 to 800 K, ensuring excellent electrical characteristics across a wide temperature range.

**Figure** **3**a,b and Figures S9 and S10 (Supporting Information) show the temperature‐dependent *κ*_total_ and *κ*_L_ of Pb‐doped Ge_0.065_Zr_0.02_Te_0.985_(Cu_2_Te)_0.015_ samples. The *κ*_total_ was measured using the laser flash method, while *κ*_L_ was determined by subtracting the carrier component (*κ*_e_ = *Lσ*T). The Lorenz number (*L*) was calculated based on the SPB model's approximation with acoustic scattering, with further details provided in the Supporting Information. Compared to the intrinsic Ge_0.98_Zr_0.02_Te sample, the *κ*_total_ of Cu_2_Te alloyed and Pb‐doped GeTe samples decreases sharply, particularly at low temperatures (e.g., from ≈3.81 to ≈1.38 W m^−1^ K^−1^ at 300 K). This reduction is primarily due to significant decreases in both *κ*_L_ and *κ*_e_. Furthermore, with increasing Pb doping, *κ*_L_ shows a substantial overall reduction, as shown in Figure 3b. Specifically, the room‐temperature *κ*_L_ decreased from ≈1.81 to 0.95 W m^−1^ K^−1^, representing a remarkable 50% reduction. Notably, *κ*_L_ reaches a minimum of ≈0.38 W m^−1^ K^−1^ at 650 K in the Ge_0.885_Zr_0.02_Pb_0.08_Te_0.985_(Cu_2_Te)_0.015_ sample, which is comparable to the amorphous limit of GeTe as calculated by the Cahill model (≈0.3 W m^−1^ K^−1^).^[^ ^35^, ^70^ ^]^ Typically, point defects from elemental alloying are recognized as key factors influencing the reduction in *κ*_L_, primarily due to strong phonon scattering caused by the size and mass differences between the matrix atoms. However, the sound velocity measurements in this work (Figure 3c) indicate that, as foreign atom doping increases, the material's average sound velocity (*v*_g_) and transverse velocity (*v*_T_) gradually decrease, while the longitudinal velocity (*v*_L_) remains unchanged. Specifically, *v*_T_ significantly decreases from ≈1980 to 1820 m s^−1^ at room temperature, indicating a pronounced suppression of *v*_T_ without affecting *v*_L_. This indicates that the Pb‐doped Ge_0.065_Zr_0.02_Te_0.985_(Cu_2_Te)_0.015_ samples exhibit a highly disordered structure, which significantly contributes to the reduction in *κ*_L_.^[^ ^71^, ^72^ ^]^ The Gruneisen parameter (γ) increases significantly from 1.4 to 1.8 (Figure S10, Supporting Information),^[^ ^60^ ^]^ which is closely proportional to the ratio of *v*_L_/*v*_T_, reflecting enhanced anharmonic phonon scattering behavior. As temperature rises, phonon scattering intensifies, and the phonon relaxation time decreases, leading to extremely low *κ*_L_ at high temperatures. This confirms that the alloying process alters the lattice periodicity of the GeTe samples, introducing the lattice disorder that precisely modifies phonon dispersion relations. These findings highlight the need for further exploration of the pronounced phonon scattering mechanisms during alloying, driven by complex microstructural centers.

**Figure 4:**
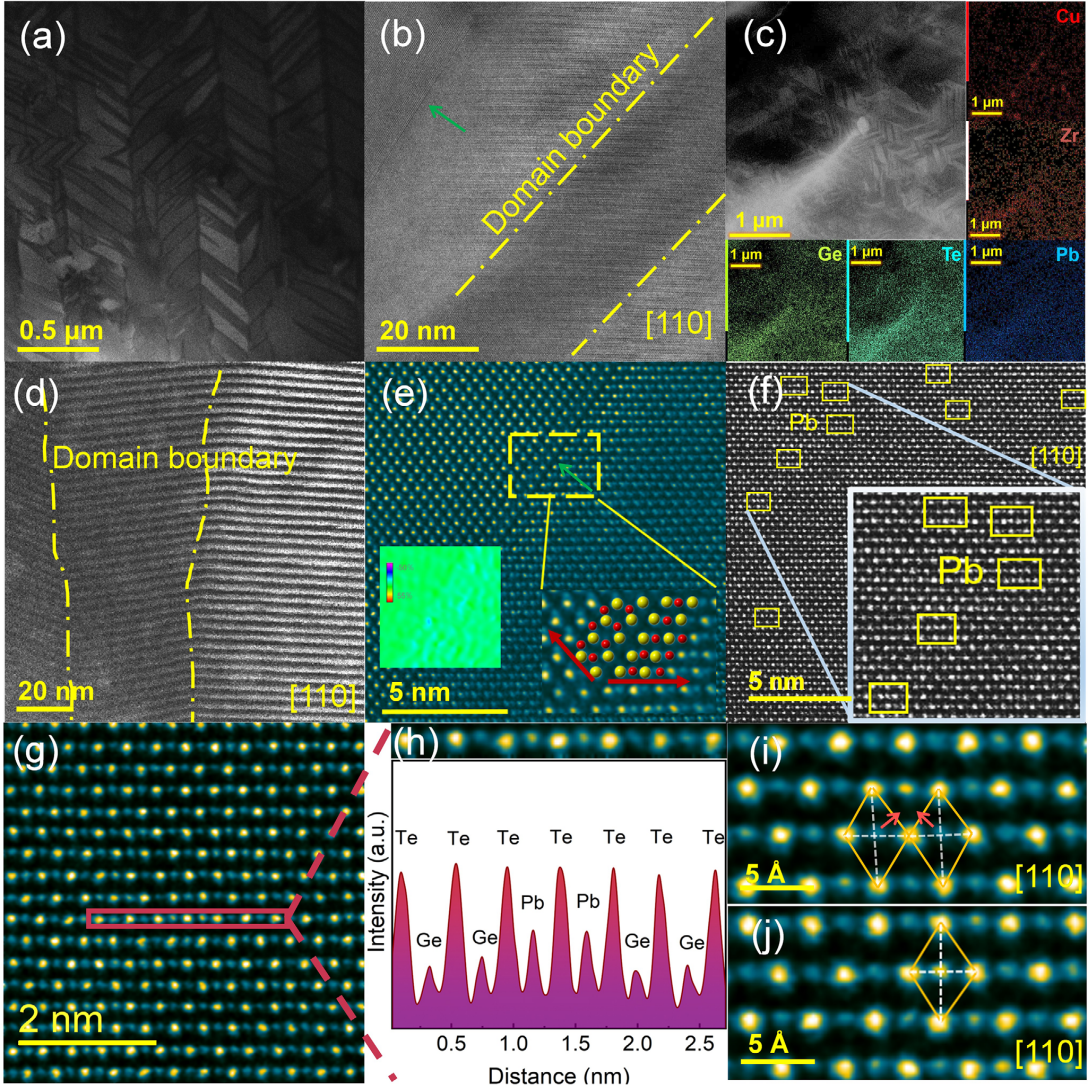
Microstructures of the Ge_0.885_Zr_0.02_Pb_0.08_Te_0.985_(Cu_2_Te)_0.015_ sample. a) Low‐magnification ABF‐STEM image showing the herringbone microstrip pattern. b) Enlarged view of the domain within the region. c) HAADF‐STEM image and EDS elemental mapping of Ge, Te, Pb, Zr, and Cu. d) Scanning moiré fringes (SMFs) image formed by the aliasing effect between the electron probe and the atomic lattice. e) Atomic‐resolution HAADF‐STEM image revealing vdW gaps, with the inset showing GPA strain mapping. f,g) Atomic substrates with abnormal contrast in the matrix, indicating irregular Pb atom distribution. h) Corresponding line intensity distribution of the atomic substrates. i) The rhombus highlights the Te atoms, with lower‐intensity Ge atoms indicated. j) Pb‐substituted Ge sites show higher intensity and occupy a more favorable geometric symmetry center, with the arrowhead marking the direction of atomic displacement.

We explored the microstructure and phonon source characteristics of the Ge_0.885_Zr_0.02_Pb_0.08_Te_0.985_(Cu_2_Te)_0.015_ sample using a spherical aberration‐corrected scanning transmission electron microscope (Cs‐corrected STEM). **Figures** **4**a and S11a (Supporting Information) present low‐magnification annular bright field (ABF) STEM images of the sample. The observed herringbone microstrips, typical of GeTe‐based materials, result from a regular arrangement of regions with contrasting polarities within black and white domains, all aligned with the same crystallographic orientation of the rhombohedral phase (Figure S11b, Supporting Information).^[^ ^73^ ^]^ Only a few Ge vacancy layers were observed within these domains in the magnified region, indicating that the formation of Ge vacancies is effectively suppressed (Figure 4b; Figure S12, Supporting Information). At the grain boundaries, nanoscale precipitates were observed, with energy dispersive spectroscopy (EDS) analysis confirming the composition of these inclusions. Elemental mapping (Figure 4c) reveals a uniform distribution of constituent elements across the microstructure, with a pronounced accumulation of Cu in certain localized areas. Higher magnification images (Figure S13, Supporting Information) confirm that these precipitates are Cu‐ and Te‐rich nanophases, characterized by distinct microstructures that effectively impede phonon transport. The scanning moiré fringes (SMFs) in Figure 4d were employed to pinpoint the positions of domain boundaries. Further magnification (Figure S14, Supporting Information) shows substantial atomic misalignments, with domain orientations differing by ≈3° and ≈0.47°, indicating significant localized stress at the nanoscale low‐angle grain boundaries (LAGB).^[^ ^73^ ^]^ A characteristic 2D defect van der Waals (vdW) gap is observed, which forms as a result of strain relief during the cubic‐to‐rhombohedral phase transition in GeTe. This gap features an ordered arrangement of Ge vacancies (Figure 4e; Figure S15, Supporting Information).^[^ ^33^ ^]^ Geometric phase analysis (GPA) suggests that these strain fluctuations can effectively scatter phonons across a broad frequency range without compromising carrier transport (Figure 4e inset; Figure S16, Supporting Information).^[^ ^45^ ^]^

Further analysis of atomic sites with unusual contrast within the matrix (Figure 4f,g; Figure S17a, Supporting Information) and corresponding intensity distribution maps (Figure 4h) indicate effective substitution of Ge atoms by heavier atoms. Atomic mass analysis shows that while Pb atoms are distributed irregularly, they preserve the intrinsic GeTe lattice structure. High‐angle annular dark‐field (HAADF) images along the standard [110] crystallographic direction (Figures 4i,j) show the typical arrangement where the brighter Te sites and the lower‐intensity Ge sites (Figure 4i) exhibit a slight deviation of Ge atoms from the rhombohedral geometric center formed by Te atoms, consistent with previous reports.^[^ ^36^ ^]^ This deviation induces a polar electric field, which in turn leads to carrier localization and reduced mobility. Interestingly, Pb‐substituted Ge sites display increased intensity and occupy a more symmetric geometric center (Figure 4j; Figure S17b, Supporting Information), resulting in enhanced phonon scattering. However, this improvement in lattice symmetry also brings about more uniform bond lengths and a consistent electronic potential, thereby promoting delocalized electron transport. Thus, the structural symmetry enhancement induced by Pb doping emerges as a critical factor in maintaining the high mobility observed in the Ge_0.885_Zr_0.02_Pb_0.08_Te_0.985_(Cu_2_Te)_0.015_ sample.

Based on HAADF‐STEM observations, we identified various multiscale defects, including point defects (PD), LAGBs, nanoprecipitates (NP), stacking faults (SFs), and domain boundaries (DB). These defects contribute to multiple phonon scattering mechanisms, encompassing intrinsic and Umklapp (U) processes, as well as normal (N) processes, along with additional scattering sources such as grain boundaries (GB). As illustrated in Figure 3d, we explored the phonon scattering behavior associated with these defects. Using the Debye approximation model, we quantified the relationship between the lattice thermal conductivity spectrum (*κ*_s_) and phonon frequency (*ω*) for each scattering mechanism in the Ge_0.885_Zr_0.02_Pb_0.08_Te_0.985_(Cu_2_Te)_0.015_ sample at 300 K, as shown in Figure 3e,f. The analysis reveals that defects such as GB, SF, DB, NP, and PD scatter phonons effectively over a wide frequency range. The presence of these multiscale defects, combined with substantial lattice distortion, induces extensive phonon scattering and softening, contributing to the exceptionally low *κ*_L_ of ≈0.38 W m^−1^ K^−1^ at 650 K.

**Figure 5:**
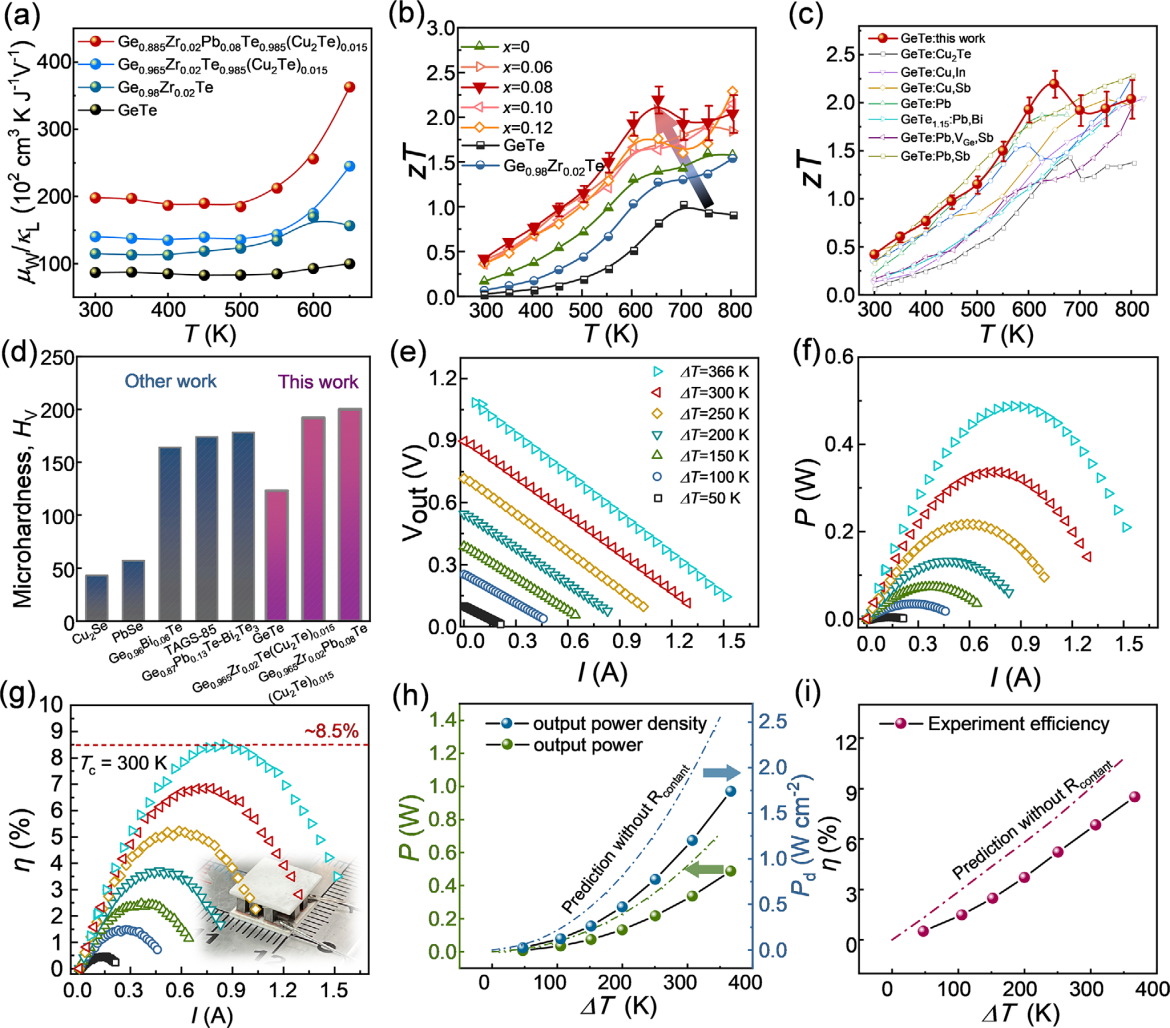
The *zT* and mechanical properties of the Ge_0.965‐_ *_x_* Zr_0.02_Pb*_x_* Te_0.985_(Cu_2_Te)_0.015_ samples, along with the TE performance of a 7‐pair device. Temperature‐dependent a) *µ*_w_/*κ*_L_ b) *zT* values with GeTe, Ge_0.98_Zr_0.02_Te, Ge_0.965_Zr_0.02_Te_0.985_(Cu_2_Te)_0.015_, and Ge_0.885_Zr_0.02_Pb_0.08_Te_0.985_(Cu_2_Te)_0.015_ in this work. c) Comparison of the *zT* of Ge_0.885_Zr_0.02_Pb_0.08_Te_0.985_(Cu_2_Te)_0.015_ with literature values for superior GeTe materials.^[^ ^40^, ^42^, ^61^, ^63^, ^74^, ^75^, ^76^ ^]^ d) Microhardness of the samples compared with other reported GeTe materials.^[^ ^32^, ^77^, ^78^, ^79^ ^]^ e) Voltage (*V*), f) output power (*P*), and g) conversion efficiency (*η*) of the device as a function of measured current at various temperature differences (Δ*T*) (with *T*_c_ = 300 K). h) Prediction of *P* and output power density (*P*_d_) without *R*_contant_ versus experiment values (with *T*_c_ = 300 K). i) Maximum conversion efficiency (*η*_max_) under different temperature gradients for the GeTe/PbSe device.

Ultimately, Pb doping introduces multiscale defects and lattice disorder, which significantly impede phonon transport while simultaneously optimizing the electronic band structure. This dual effect enables carriers to achieve delocalized transport, influenced by the presence of high‐symmetry geometric centers. As a result, the Ge_0.885_Zr_0.02_Pb_0.08_Te_0.985_(Cu_2_Te)_0.015_ sample demonstrates a substantial reduction in *κ*_L_ and outstanding performance in *µ*_w_ across the entire temperature range (**Figure** **5**a). This effective decoupling of carrier and phonon transport markedly improves the *µ*_w_/*κ*_L_ ratio, achieving values of ≈1.93 × 10^4^ cm^3^ K J^−1^ V^−1^ at room temperature and ≈3.63 × 10^4^ cm^3^ K J^−1^ V^−1^ at 650 K, outperforming the original Ge_0.98_Zr_0.02_Te sample. As a result, a significant enhancement in *zT* is observed over the entire temperature range. Cyclic measurements of the TE characteristics of the Ge_0.885_Zr_0.02_Pb_0.08_Te_0.985_(Cu_2_Te)_0.015_ sample indicate excellent thermal stability (Figure S18, Supporting Information). Notably, the peak *zT* reaches ≈2.2 at 650 K (Figure 5b; details provided in Table S3, Supporting Information), with a high average *zT*_ave_ of ≈1.4 over the temperature range of 300 to 800 K. When compared to the existing literature, Ge_0.885_Zr_0.02_Pb_0.08_Te_0.985_(Cu_2_Te)_0.015_ surpasses the performance of most reported high‐performance Cu‐ and Pb‐alloyed GeTe systems within the 300–800 K range (Figure 5c).^[^ ^40^, ^42^, ^61^, ^63^, ^74^, ^75^, ^76^ ^]^ In addition to its exceptional TE performance, the Vickers microhardness significantly increases from ≈123 *H*_v_ in pristine GeTe to ≈200 *H*_v_ in the Ge_0.885_Zr_0.02_Pb_0.08_Te_0.985_(Cu_2_Te)_0.015_ sample. This notable improvement in hardness is attributed to its enriched microstructure, which plays a crucial role in crack suppression, making it one of the best hardness values reported for GeTe‐based and other typical TE materials (Figure 5d).^[^ ^32^, ^77^, ^78^, ^79^ ^]^

To further demonstrate the practical potential of our GeTe‐based TE material, we fabricated a 7‐pair device using *p*‐type Ge_0.885_Zr_0.02_Pb_0.08_Te_0.985_(Cu_2_Te)_0.015_ and *n*‐type PbSe samples (Figure S19, Supporting Information), with Cu as the electrode and a thin Ni bonding layer. Figure 5e–g and Figure S20 (Supporting Information) illustrate the relationships between output voltage (*V*), output power (*P*), conversion efficiency (*η*), and heat flow (*Q*_c_) as functions of current (*I*) under different Δ*T*. The device achieved a maximum output power (*P*) of 0.5 W, resulting in an output power density (*P*_d_ = *P*/*A*) of ≈1.7 W cm^−2^ (Figure S21, Supporting Information). The maximum *η*_max_ reached ≈8.5% at Δ*T* = 366 K, with the cold side temperature fixed at 300 K. The device exhibited excellent stability during throughout the testing process (Figure S22, Supporting Information). By optimizing the multistage device bonding process to minimize the gap between experimental characteristic internal resistance (*R*_in_) and theoretical values, we simulated an enhanced output power of ≈0.7 W, corresponding to a power density of ≈2.6 W cm^−2^ (with *T*_cold_ = 300 K). This optimization also led to a competitive theoretical conversion efficiency of ≈10.8% for the 7‐pair device at Δ*T* = 350 K, not accounting for the impact of *R*_in_ (Figure 5h,i).

## 3 Conclusion

In conclusion, we have significantly enhanced the TE properties of GeTe‐based materials. Through systematic theoretical calculations and precise structural characterizations, we demonstrated that the optimized band structure and enhanced crystal symmetry led to delocalized electron transport and a high *µ*_w_, thereby achieving an exceptional *PF*. The incorporation of multiscale defects and substantial lattice disorder effectively modified phonon dispersion, reducing *κ*_L_ to ≈0.38 W m^−1^ K^−1^ at 650 K. This optimization greatly improves the *µ*_w_/*κ*_L_ ratio in the Ge_0.885_Zr_0.02_Pb_0.08_Te_0.985_(Cu_2_Te)_0.015_ sample, which also demonstrates exceptional Vickers microhardness (≈200 *H*_v_) and superior TE performance, achieving a peak *zT* of ≈2.2 at 650 K and an average *zT*_ave_ of ≈1.4 from 300 to 800 K, ensuring both reliability and practicality in device applications. Moreover, the fabricated 7‐pair device showcased an impressive maximum *η*_max_ of ≈8.5% and a high *P*_d_ of ≈1.7 W cm^−2^ at Δ*T* = 366 K, confirming its strong potential for real‐world applications. This work underscores the pivotal role of band‐crystal engineering in weakening carrier‐phonon coupling, paving the way for the development of high‐performance TE materials and offering valuable insights for designing advanced TE devices.

## Conflict of Interest

The authors declare no conflict of interest.

## Supporting information



Supporting Information

## Data Availability

The data that support the findings of this study are available from the corresponding author upon reasonable request.
