# Supplementary material for: Enhanced Band‐Crystal Engineering Drives Superior Power Generation in GeTe
Source: Adv Sci (Weinh). 2025 May 22;12(31):e06612. doi: 10.1002/advs.202506612 (PMC12376500; doi:10.1002/advs.202506612)
Supplement: Supplementary file 1 — Supporting Information [file ADVS-12-e06612-s001.docx]

**Supplementary materials**

**Enhanced Band-Crystal Engineering Drives Superior Power Generation in GeTe**

Xiaobo Tan, Qian Deng, Jianglong Zhu, Ruiheng Li, Xuri Rao, Fan Feng, Shuang Lyu, Pengfei Nan* Yue Chen, Binghui Ge, Ran Ang*

X.B. Tan, Q. Deng, J.L. Zhu, R.H. Li, X.R. Rao, F. Feng, R. Ang

Key Laboratory of Radiation Physics and Technology, Ministry of Education, Institute of Nuclear Science and Technology, Sichuan University, Chengdu 610064, China

S. Lyu, Y. Chen

Department of Mechanical Engineering, The University of Hong Kong, Pokfulam Road, Hong Kong SAR, China

B.H. Ge, P.F. Nan

Information Materials and Intelligent Sensing Laboratory of Anhui Province, Leibniz International Joint Research Center of Materials Sciences of Anhui Province, Institutes of Physical Science and Information Technology, Anhui University, Hefei 230601, China

R. Ang

College of Physics, Sichuan University, Chengdu 610064, China

Institute of New Energy and Low-Carbon Technology, Sichuan University, Chengdu 610065, China

*Correspondence and requests for materials should be addressed to R.A. (email: [rang@scu.edu.cn)](mailto:rang@scu.edu.cn)) or P.F.N. (email: [npf@ahu.edu.cn)](mailto:npf@ahu.edu.cn))

(Xiaobo Tan and Qian Deng contribute equally to this work)

Keywords: thermoelectric, conversion efficiency, GeTe, band engineering, crystal structure

1. **Experiment details**
   1. **Sample synthesis:** High-purity elements [Ge (99.999%), Te (99.999%), Zr (99.95%), Cu_2_Te (99.99%), Pb (99.999%)] were sealed in evacuated quartz tubes and melted at 1123 Κ for 8 hours. The molten mixture was rapidly quenched in ice water and subsequently annealed at 903 Κ for 72 hours to synthesize polycrystalline samples of the composition Ge_0.985-_*_x_*_-_*_y_*Zr*_y_*Pb*_x_*Te_0.985_(Cu_2_Te)_0.015_ (with *y*= 0, 0.01, 0.02, 0.03; *x*= 0.04, 0.06, 0.08, 0.1, 0.12). The resulting ingots were ground into fine powder for X-ray diffraction (XRD) analysis and rapid hot pressing (HP). Hot pressing was performed using an induction heating system at 823 Κ for 40 minutes under a uniaxial pressure of ~75 MPa. The final specimens, with a diameter of 12.5 mm, achieved a density of at least 98% of the theoretical value, as determined by the Archimedes method (**Table S1**). High-purity starting materials were precisely weighed according to stoichiometric amounts for the synthesis of polycrystalline *n*-type PbSe. The raw materials were sealed in evacuated quartz tubes (<10^–3^Pa), heated to 1423 K over 6.5 hours, held at this temperature for 8 hours, then quenched to room temperature and annealed at 923 K for 100 hours. The resulting PbSe ingots were finely ground into powder using an agate mortar and subsequently sintered by hot pressing at an axial pressure of 55 MPa and 873 K for 0.5 hours. This process yielded high-density cylindrical samples with a diameter of ~12.7 mm.
   2. **Structural characterization:** Powder X-ray diffraction (XRD) measurements were performed using Cu–Kα (λ =1.5418 Å) radiation on an X-ray diffractometer. The morphological, atomic structural, and chemical composition of the synthesized samples were analyzed using a JEOL-NEOARM200F transmission electron microscope (200 kV), microscope equipped with a probe corrector and energy dispersive spectroscopy (EDS).
   3. **Transport property measurements:** The electrical conductivity (σ) and Seebeck coefficient (S) were measured using a CTApro measurement system (Beijing Cryoall Science and Technology Co., Ltd. China). The total thermal conductivity (*κ*_total_) was calculated using the formula *κ*_total_ = *ρC*_p_*D*, where *ρ* is the density, *C*_p_ is the specific heat capacity, and *D* is the thermal diffusivity. The *D* was determined via the laser flash method using a Netzsch LFA 467 instrument (Netzsch, Shanghai, China). Hall measurements were performed using the Van der Pauw technique under a magnetic field of 1.5 *T*. The carrier concentration (*n*_H_) and mobility (*μ*) were calculated using *n*_H_ = 1/(*eR*) and *μ* = *σR*, where *e* is the electron charge. Sound velocities (*v*) were measured using a pulse-receiver (Olympus-NDT) in conjunction with an oscilloscope (Keysight). Microhardness (*H*_v_) was measured with a Vickers diamond indenter on the HVS-1000, with a 2 N force applied and the indent held for 10 seconds.
   4. **Density functional theory (DFT) calculations:** DFT calculations were performed using the Vienna Ab-initio Simulation Package (VASP)^[1]^ to obtain the total density of states (TDOS). The Perdew-Burke-Ernzerhof (PBE) generalized gradient approximation (GGA),^[2]^ within the projector augmented wave (PAW) method,^[3]^ was used to calculate the exchange-correlation energy. A plane-wave energy cut-off of 400 eV and a sampling spacing of 2π × 0.02 Å^-1^, with a Γ-centered Monkhorst-Pack scheme in the Brillouin zone, were adopted in all calculations. A 4×4×3 supercell (Ge_48_Te_48_) derived from the primitive cell was utilized to achieve desired doping concentrations via the Alloy-Theoretic Automated Toolkit (ATAT) package.^[4]^ All structures were fully relaxed until the force and energy converged to criteria of 10^−3^ eV/Å and 10^−6^ eV, respectively. Self-consistent total energy calculations were conducted with convergence set to 10^-8^ eV.
   5. **Thermoelectric power generation test:** A thermoelectric (TE) device with 7 pairs of *p-n* legs was fabricated using *p*-type Ge_0.885_Zr_0.02_Pb_0.08_Te_0.985_(Cu_2_Te)_0.015_ and *n*-type PbSe, with each leg having dimensions of 1.4 mm × 1.4 mm× 3.0 mm. Nickel was electroplated onto the legs as a diffusion barrier. The device assembled involved directly bonding the *p*- and *n*-type legs in series onto a copper direct-bonded copper (DBC) ceramic substrate using conductive Ag-based silver solder. The electrical conversion efficiency and power output of the TE device were evaluated using a custom-built test system, which was calibrated and validated with a Mini-PEM (Advance Riko, Japan).
2. **Details of modeling study based on single parabolic band (SPB)**

The Seebeck coefficient *S*:

$S=\frac{k_{b}}{e}[\frac{\left( r+5/2 \right)F_{r+3/2}\left( \eta\right)}{\left( r+3/2 \right)F_{r+1/2}\left( \eta\right)}-\eta]$ (S1)

where *η* is the reduced chemical potential, *k*_B_ is the Boltzmann constant, *e* is the electron charge, and *r* is the scattering factor.

The Hall carrier concentration $n_{H}$:

$n_{H}=4\pi{[\frac{{2m^{*}k}_{B}T}{h^{2}}]}^{3/2} F_{1/2}$ (S2)

where *m*^*^ is the effective mass of the density of states, accounting for band degeneracy, *h* is the Plank constant, and *T* is the absolute temperature.

The mobility *μ_H_*:

$\mu_{H}=\mu_{0}\frac{F_{-1/2}}{{2F}_{0}}=\frac{\tau_{0}e}{m^{*}}\frac{F_{-1/2}}{{2F}_{0}}$ (S3)

where $\tau_{0}$ is the relaxation time, which is closely related to the energy in the case of acoustic phonon scattering: $\tau_{0}=\frac{h^{4}C_{l}}{8\sqrt{2}\pi^{3}{E_{def}}^{2}{m^{*}kT}^{3/2}}$, where *C*_l_ is a parameter determined by the combination of the elastic constant, and *E_def_* is a combination of deformation potentials for multivalley systems.

The Hall factor *A*:

$A=\frac{3}{2}F_{1/2}\left( \eta\right)\frac{F_{{-1}/2}}{2F_{0}^{2}}$ (S4)

The Hall factor reflects the energy scattering mechanism and the anisotropy of the energy band. In the SPB model, anisotropy does not need to be considered.

The Lorenz number *L*:

$L=\frac{\kappa_{B}^{2}}{e^{2}}\frac{3F_{0}F_{2}-4F_{1}^{2}}{F_{0}^{2}}$ (S5)

In the equations above, the integral $F_{j}$ is defined by

$F_{j}\left( \eta\right)=\int_{0}^{\infty} \frac{\xi^{j}d\xi}{1+e^{(\xi-\eta)}}$ (S6)

1. **Details of calculation of lattice thermal conductivity *κ*_L_**

The *κ*_L_ of alloys was calculated using the modified Debye-Callaway model, expressed by the following equation ^[5-8]^：

${}_{L}=\frac{K_{B}}{2{}^{2}\upsilon}\left( \frac{K_{B}}{\hbar} \right)^{3}\int_{0}^{/T} \left( x \right)\frac{x^{4}e^{x}}{\left( e^{x}-1 \right)^{2}}dx$ (S7)

where the integrand, combined with the coefficients of the above equation represents the spectral lattice thermal conductivity (*κ*_s_), given by:

${}_{s}=\frac{k_{B}}{2{}^{2}\upsilon}\left( \frac{K_{B}}{\hbar} \right)^{3}\left( x \right)\frac{x^{4}e^{x}}{\left( e^{x}-1 \right)^{2}}$ (S8)

Here, $v=3^{1/3}{({v_{l}}^{-3}+2{v_{t}}^{-3})}^{-1/3}$ ($v_{l}$ and $v_{t}$ denote the transverse and longitudinal sound velocities, respectively) is the average speed of phonon, *x* = *ħω*/*k_B_T* (with *ω* denoting the phonon frequency) represents the reduced phonon frequency, is Debye temperature, *ħ* is the reduced Planck constant, $k_{B}$is the Boltzmann constant, and *τ_c_* is the total relaxation time. The combined relaxation time *τ_c_* for the Ge_0.885_Zr_0.02_Pb_0.08_Te_0.985_(Cu_2_Te)_0.015_ sample includes contributions from Umklapp processes (U), normal processes (N), grain boundaries (GB), point defects (PD), nanoprecipitates (NP), stacking faults (SF), and domain boundary (DB) as follows:

$\tau_{c}^{-1}=\tau_{U}^{-1}+\tau_{N}^{-1}+\tau_{B}^{-1}+\tau_{PD}^{-1}+\tau_{NP}^{-1}+\tau_{SF}^{-1}+\tau_{DB}^{-1}$ (S9)

Umklapp and Normal process phonon scattering:

${}_{U}^{-1}=\frac{\hbar{}^{2}{}^{2}T}{Mv^{2}}e^{-/T}$ (S10)

$\tau_{N}^{-1}=\beta\tau_{U}^{-1}$ (S11)

where $M$ is the average atomic mass, *γ* is the Grüneisen parameter, and *β* is the ratio between normal process and Umklapp phonon scattering.

GB phonon scattering is given by:

$\tau_{B}^{-1}=\frac{\nu}{d}$ (S12)

where *d* is the average grain size.

Point defect phonon scattering:

${}_{PD}^{-1}=\frac{V_{0}}{4{}^{2}\upsilon}{}^{4}$ (S13)

where *V_0_* is the average atomic volume, and Γ is the point defect scattering parameter. Disorder scattering parameter $\Gamma$ are derived from the Slack and Abeles models, assuming $\Gamma=\Gamma_{M}+\Gamma_{S}$, where the scattering parameters $\Gamma_{M}$ and $\Gamma_{S}$ are due to mass and strain field fluctuations, respectively. The mass and strain fluctuation scattering parameter is given by:

$\Gamma_{M}=\frac{\sum_{i=1}^{n} {c_{j}({\bar{M_{i}}}/M)}^{2}f_{i}^{1}f_{i}^{2}{[{(M_{i}^{1}-M_{i}^{2})}/{\bar{M_{i}}}]}^{2}}{\sum_{i=1}^{n} c_{i}}$ (S14)

$\Gamma_{S}=\frac{\sum_{i=1}^{n} {c_{j}({\bar{M_{i}}}/M)}^{2}f_{i}^{1}f_{i}^{2}\varepsilon{[{(r_{i}^{1}-r_{i}^{2})}/{\bar{r_{i}}}]}^{2}}{\sum_{i=1}^{n} c_{i}}$ (S15)

where *n*, *c*_j_, $\bar{M_{i}}$, $M$, $M_{i}^{k}$,$r_{i}^{k}$, $f_{i}^{k}$ and *ε* are the number of sublattice, relative degeneracies, average atomic masses, average atomic mass of the compound, atomic mass of the *k*_th_ atom of the *i_th_* sublattice, the atomic radius of the *k*_th_ atom of *i_th_* sublattice, fractional concentrations of *k_th_* atom of the *i_th_* sublattice, and lattice inharmonic parameter, respectively.

Nanoprecipitates scattering:

${}_{NP}^{-1}=\upsilon{(\left( 2\pi R^{2} \right)^{-1}+\left( \frac{4}{9}\pi R^{2}\left( \frac{\Delta D}{D} \right)^{2}\left( \frac{\omega R}{\nu} \right)^{4} \right)^{-1})}^{-1}N_{p}$ (S16)

where *R* and *D* are the average radius and density of nanoprecipitates, respectively, and *ΔD* is the density difference between the precipitated phase and the matrix material, and *N_P_* is the density of nanoprecipitates.

SF phonon scattering is given by^[9]^:

$\tau_{SF}^{-1}=0.7\frac{a^{2}}{v}\gamma^{2}\omega^{2}N_{s}$ (S17)

where *a* is the lattice parameter, and *N*_s_ is the number of SF crossing a unit length.

DB scattering is described by ^[10]^:

$\tau_{DB}^{-1}=A\frac{\nu}{d_{DB}}$ (S18)

where *A* is the domain fitting parameter, and *d*_BD_ is the average domain width.

The parameters required for the Debye-Callaway model are summarized in Table S2.


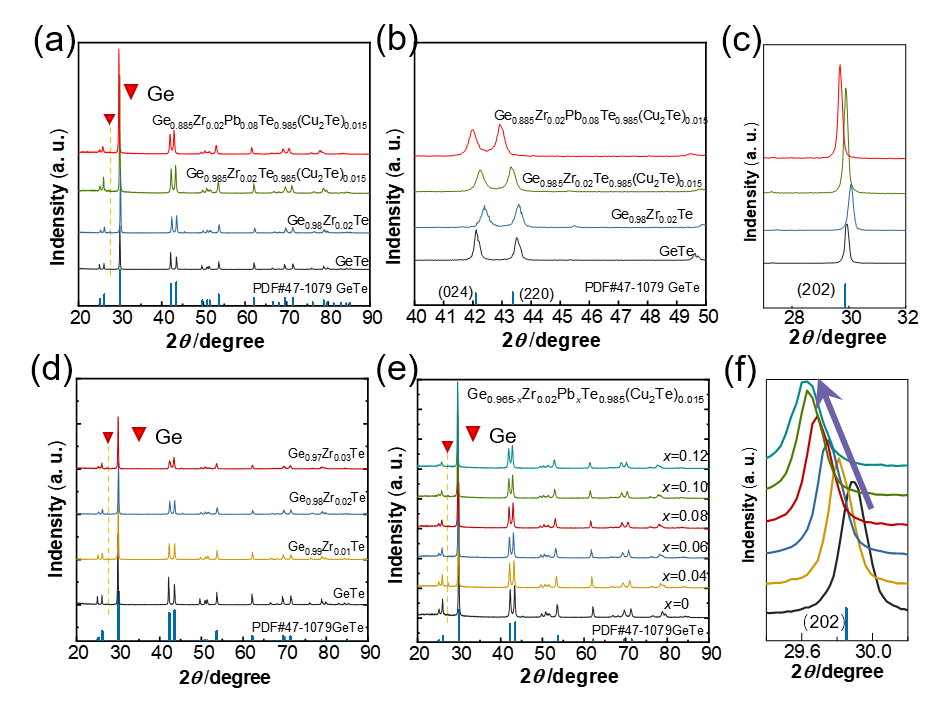


**Figure S1**. XRD patterns of the Zr-, Cu_2_Te-, and Pb-doped GeTe samples. (a) Room-temperature powder XRD patterns for the Zr-, Cu_2_Te-, and Pb-doped GeTe samples, including (d) XRD patterns for Ge_0.99_Zr_0.01_Te, Ge_0.98_Zr_0.02_Te, and Ge_0.97_Zr_0.03_Te, as well as (e) Ge_0.965-_*_x_*Zr_0.02_Pb*_x_*Te_0.985_(Cu_2_Te)_0.015_ (*x* = 0-0.12). (b) Enlarged view of the (024) and (220) peaks. (c) Enlarged view of the (202) peak for Zr-, Cu_2_Te-, and Pb-doped GeTe samples. (f) Enlarged view of the (202) peak for Ge_0.965-_*_x_*Zr_0.02_Pb*_x_*Te_0.985_(Cu_2_Te)_0.015_ (*x* = 0-0.12).


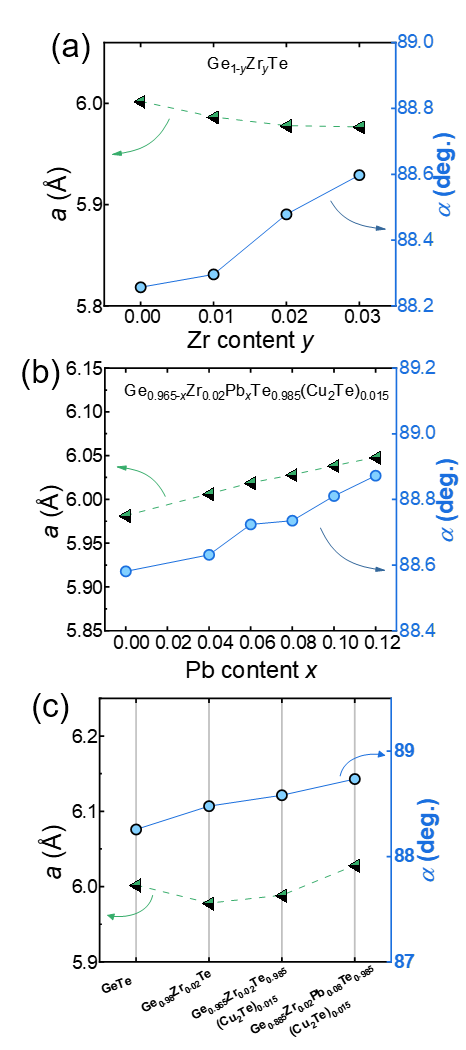


**Figure S2**. Composition-dependent lattice parameters and interaxial angles. (a) Lattice parameters and interaxial angles for Ge_0.99_Zr_0.01_Te, Ge_0.98_Zr_0.02_Te, and Ge_0.97_Zr_0.03_Te. (b) Lattice parameters and interaxial angles for Ge_0.965-_*_x_*Zr_0.02_Pb*_x_*Te_0.985_(Cu_2_Te)_0.015_ (*x* = 0-0.12). (c) Lattice parameters and interaxial angles for Zr-, Cu_2_Te-, and Pb-doped GeTe samples.


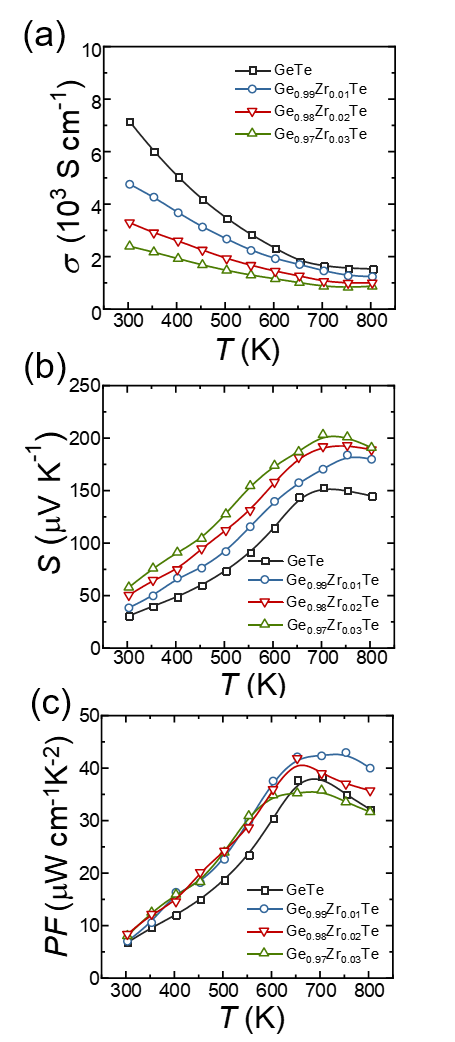


**Figure S3**. Temperature-dependent electrical properties. (a) Electrical conductivity *σ*, (b) Seebeck coefficient *S*, and (c) Power factor *PF* for GeTe, Ge_0.99_Zr_0.01_Te, Ge_0.98_Zr_0.02_Te, and Ge_0.97_Zr_0.03_Te.


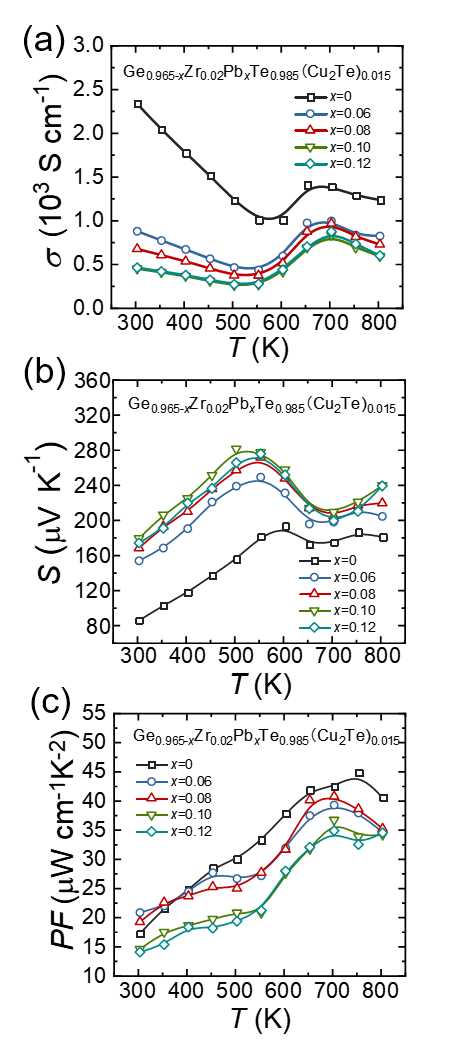


**Figure S4**. Temperature-dependent electrical properties. (a) Electrical conductivity *σ*, (b) Seebeck coefficient *S*, and (c) Power factor *PF* for Ge_0.965-_*_x_*Zr_0.02_Pb*_x_*Te_0.985_(Cu_2_Te)_0.015_ (*x* = 0-0.12) samples.


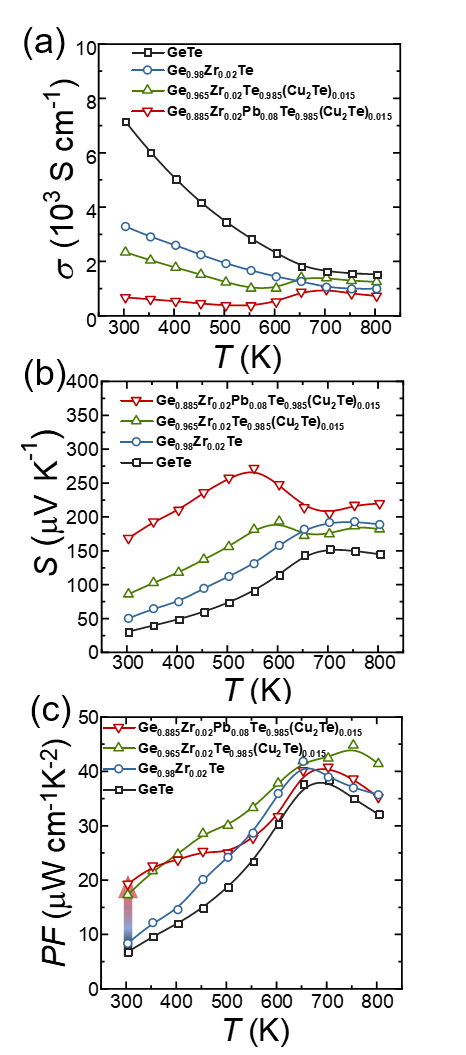


**Figure S5**. Temperature-dependent electrical properties. (a) Electrical conductivity *σ*, (b) Seebeck coefficient *S*, and (c) Power factor *PF* for for Zr-, Cu_2_Te-, and Pb-doped GeTe samples.


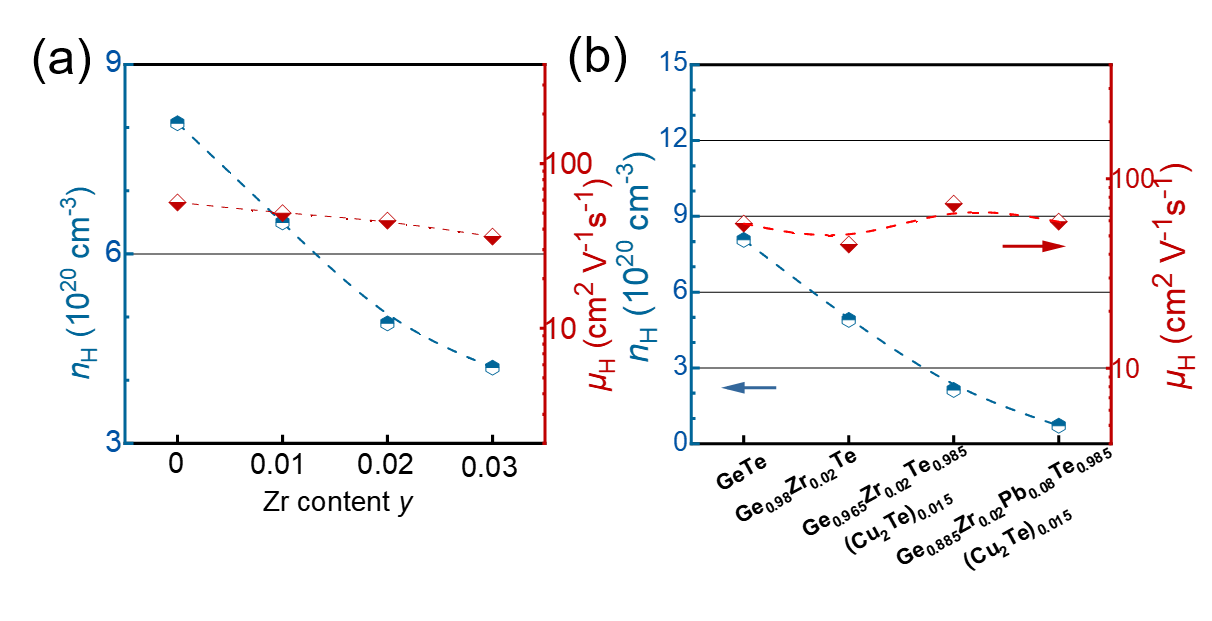


**Figure S6**. Carrier concentration *n*_H_ and carrier mobility *μ*_H_ for (a) Lattice parameters and interaxial angles for Ge_0.99_Zr_0.01_Te, Ge_0.98_Zr_0.02_Te, and Ge_0.97_Zr_0.03_Te, and (b) Zr-, Cu_2_Te-, and Pb-doped GeTe samples.


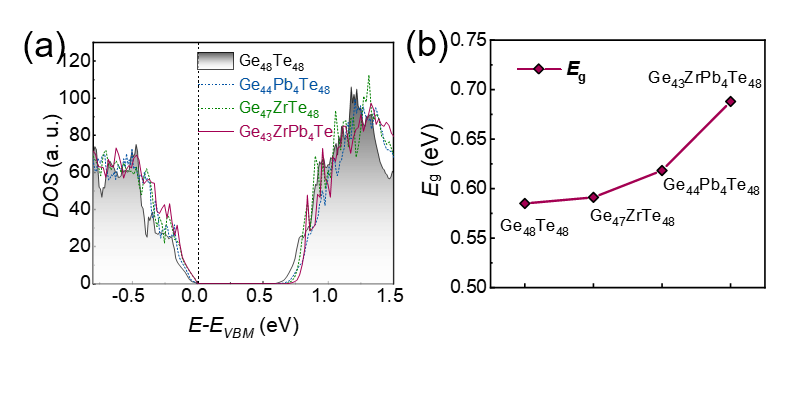


**Figure S7**. (a) Calculated total density of states (TDOS), (b) Bandgap values for pristine rhombohedral Ge_48_Te_48_, Ge_47_ZrTe_48_, Ge_44_Pb_4_Te_48_, and Ge_43_Pb_4_ZrTe_48_. The valence band maximum (VBM) is set to 0 eV.


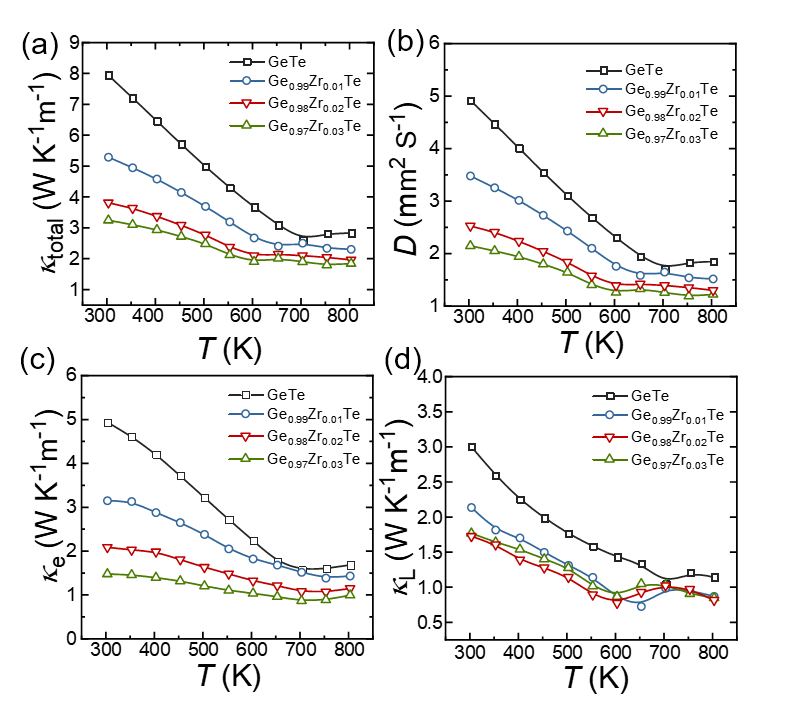


**Figure S8**. Temperature-dependent (a) total thermal conductivity (*κ*_total_), (b) thermal diffusivity (*D*), (c) electronic thermal conductivity (*κ*_e_), and (d) lattice thermal conductivity (*κ*_L_) for GeTe, Ge_0.99_Zr_0.01_Te, Ge_0.98_Zr_0.02_Te, and Ge_0.97_Zr_0.03_Te.


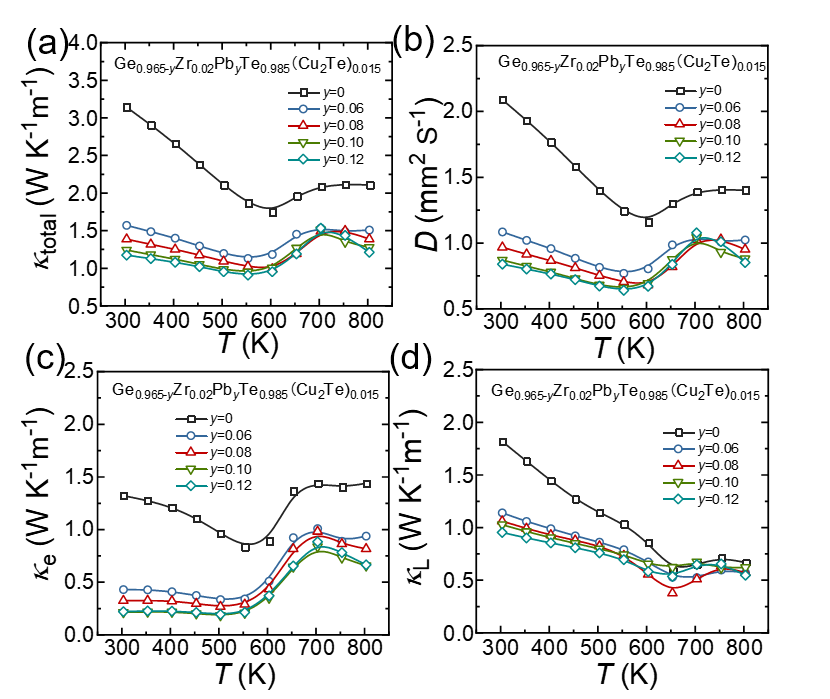


**Figure S9**. Temperature-dependent (a) total thermal conductivity (*κ*_total_), (b) thermal diffusivity (*D*), (c) electronic thermal conductivity (*κ*_e_), and (d) lattice thermal conductivity (*κ*_L_) for Ge_0.965-_*_x_*Zr_0.02_Pb*_x_*Te_0.985_(Cu_2_Te)_0.015_ (*x* = 0-0.12) samples.


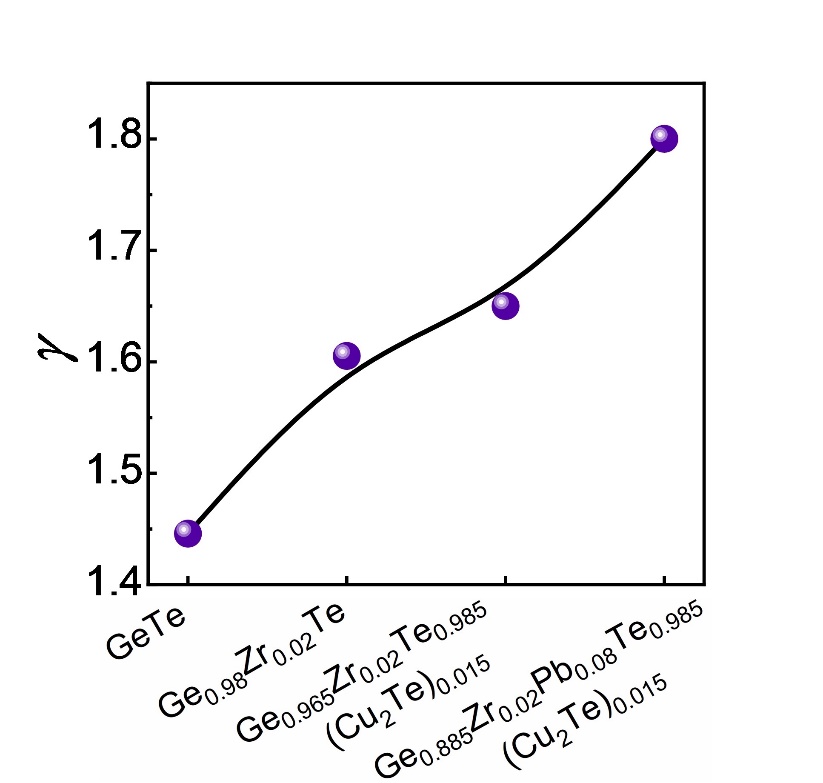


**Figure S10**. Calculated Gruneisen parameter (*γ*) for Zr-, Cu_2_Te-, and Pb-doped GeTe samples.


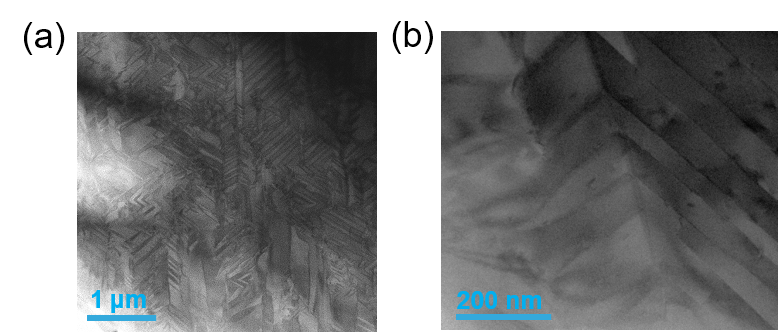


**Figure S11**. (a) Low-magnification and (b) medium-magnification ABF-STEM image showing the herringbone microstripes in the Ge_0.885_Zr_0.02_Pb_0.08_Te_0.985_(Cu_2_Te)_0.015_ sample.


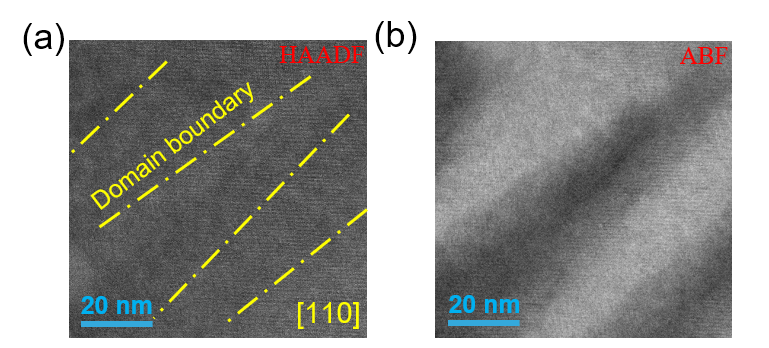


**Figure S12**. (a) High-magnification HAADF-STEM images showing enlarged domains, and (b) the contrast of these domains in the corresponding ABF image, clearly visible in the Ge_0.885_Zr_0.02_Pb_0.08_Te_0.985_(Cu_2_Te)_0.015_ sample.


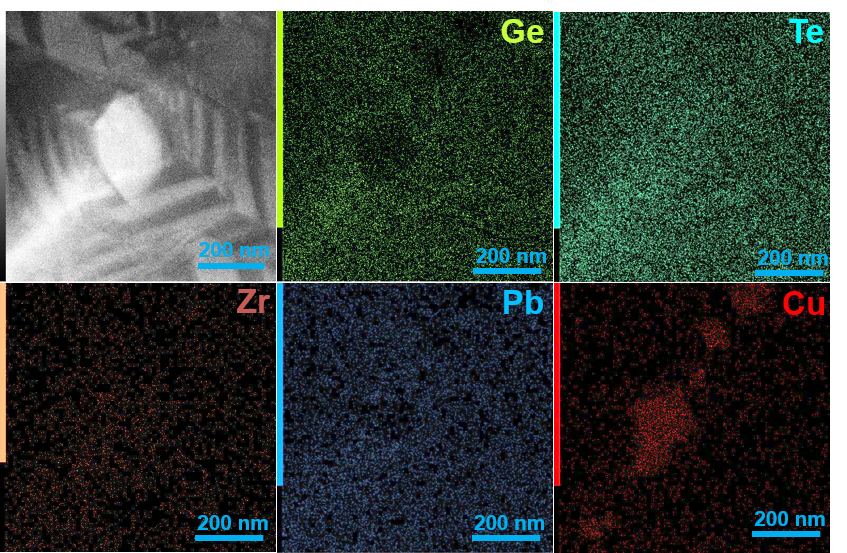


**Figure S13**. HAADF-STEM image and EDS elemental mapping of Ge, Te, Pb, Zr, and Cu within the Ge_0.885_Zr_0.02_Pb_0.08_Te_0.985_(Cu_2_Te)_0.015_ sample.


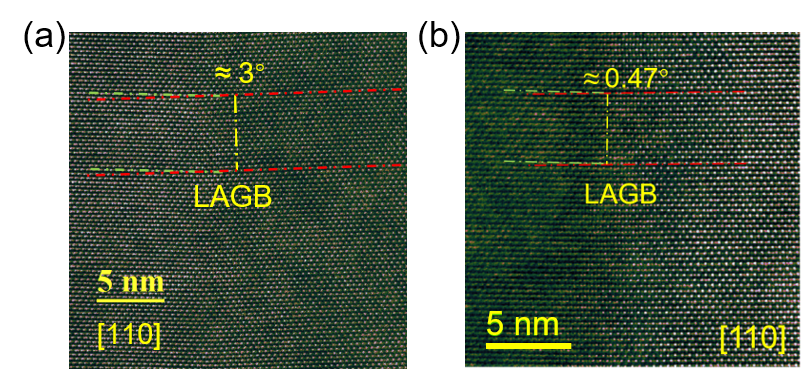


**Figure S14**. Atomic-resolution HAADF-STEM images of the low-angle grain boundary (LAGB) in the Ge_0.885_Zr_0.02_Pb_0.08_Te_0.985_(Cu_2_Te)_0.015_ sample, showing angle deviations of (a) ~3° and (b) ~0.47°.


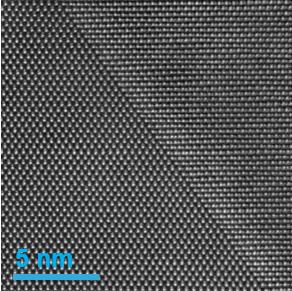


**Figure S15**. Atomic-resolution HAADF-STEM image revealing vdW gaps in the Ge_0.885_Zr_0.02_Pb_0.08_Te_0.985_(Cu_2_Te)_0.015_ sample.


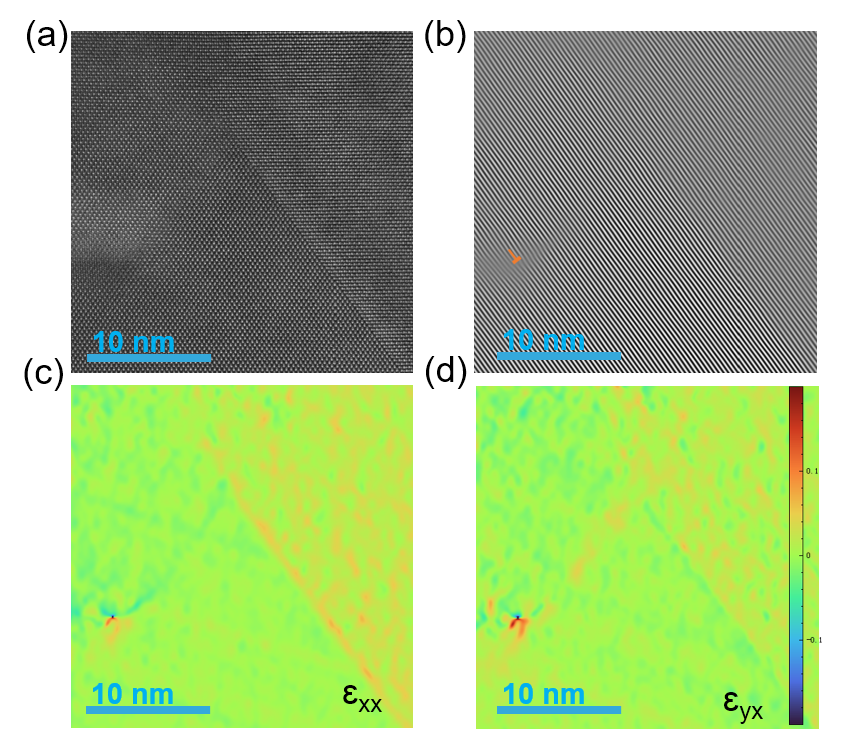


**Figure S16**. (a) High-magnification HAADF-STEM image showing vdW gaps, (b) corresponding inverse fast Fourier transform (IFFT) image of the selected area, and (c,d) corresponding geometric phase analysis (GPA) strain mapping of the selected area in (a) for the Ge_0.885_Zr_0.02_Pb_0.08_Te_0.985_(Cu_2_Te)_0.015_ sample.


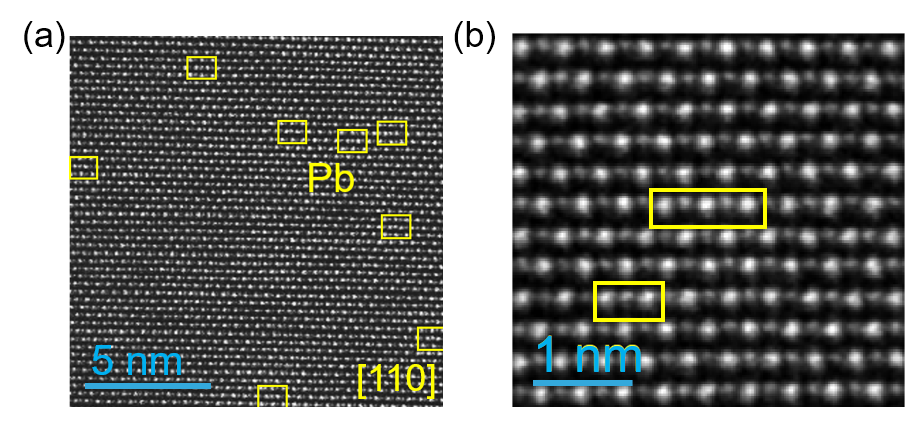


**Figure S17**. (a) Atomic-resolution HAADF-STEM image and (b) magnified image showing atomic substrates with abnormally different contrasts in the matrix, revealing that Pb atoms exhibit an irregular distribution within the Ge_0.885_Zr_0.02_Pb_0.08_Te_0.985_(Cu_2_Te)_0.015_ sample.


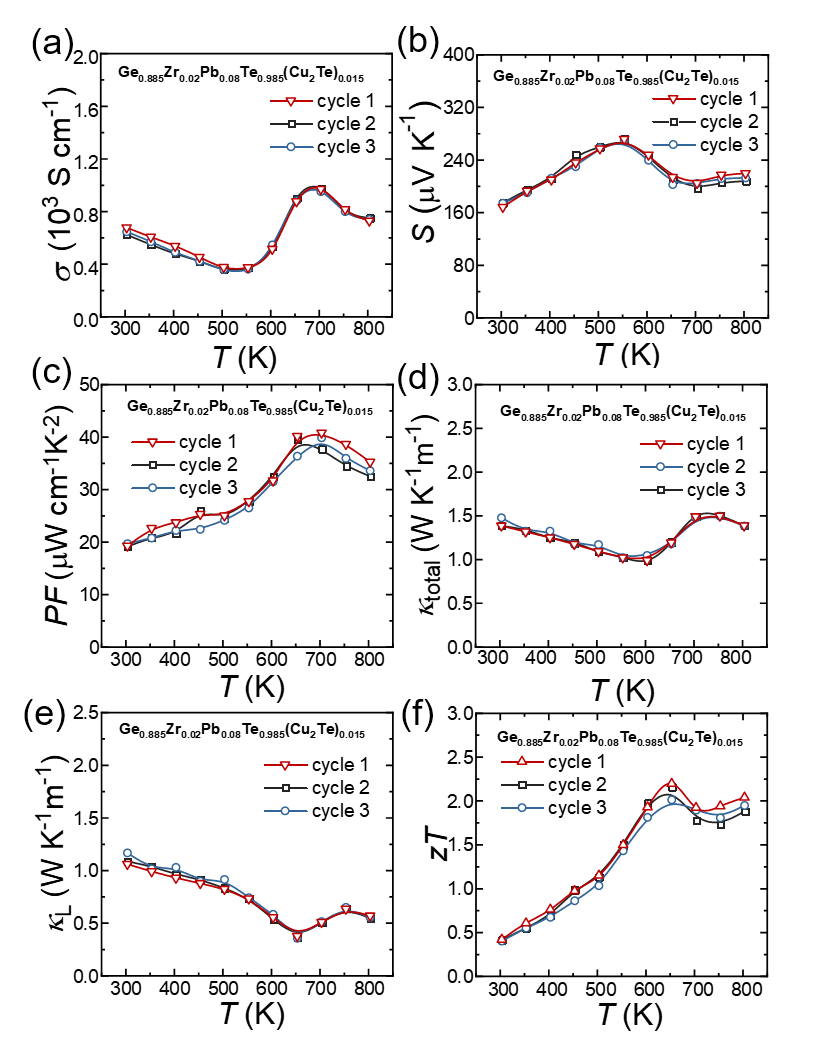


**Figure S18**. Repeatability and thermal stability of the thermoelectric transport properties in Ge_0.885_Zr_0.02_Pb_0.08_Te_0.985_(Cu_2_Te)_0.015_. (a) Electrical conductivity *σ*, (b) Seebeck coefficient *S*, (c) power factor *PF*, (d) total thermal conductivity *κ*_total_, (e) lattice thermal conductivity *κ*_L_, and (f) figure-of-merit *zT*.


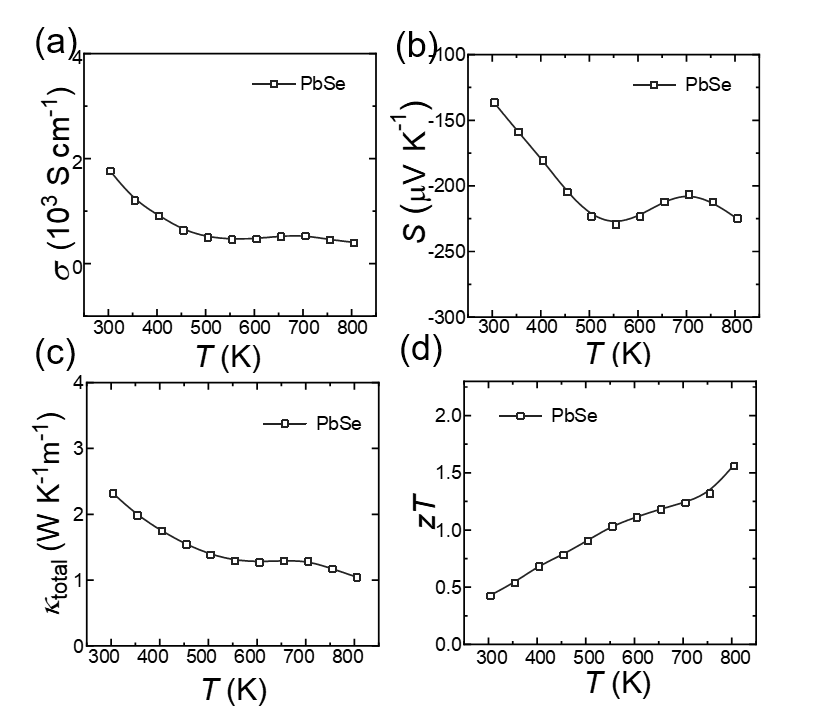


**Figure S19**. Thermoelectric performance of *n*-type PbSe for single-stage device construction. (a) Electrical conductivity *σ*, (b) Seebeck coefficient *S*, (c) total thermal conductivity *κ*_total_, and (d) figure-of-merit *zT*.


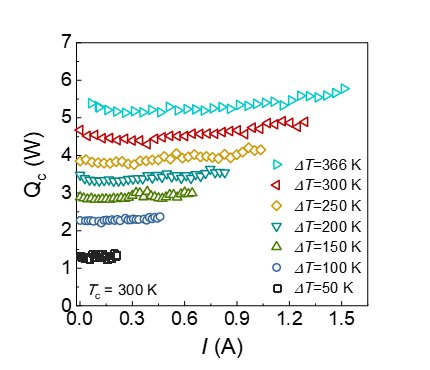


**Figure S20**. Heat flow for a 7-pair device based on the Ge_0.885_Zr_0.02_Pb_0.08_Te_0.985_(Cu_2_Te)_0.015_ sample.


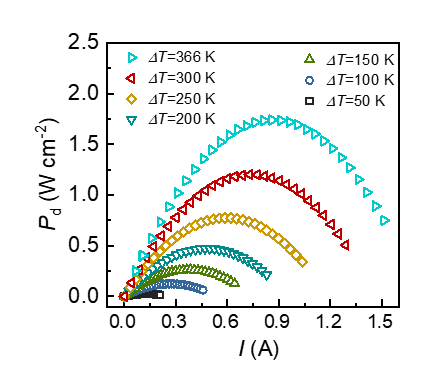


**Figure S21**. (a) Current-dependent power density (*P*_d_) for 7-pair device based on the Ge_0.885_Zr_0.02_Pb_0.08_Te_0.985_(Cu_2_Te)_0.015_ sample. (b) Internal resistance (*R*_in_), with curves showing predictions based on the thermoelectric material's properties.
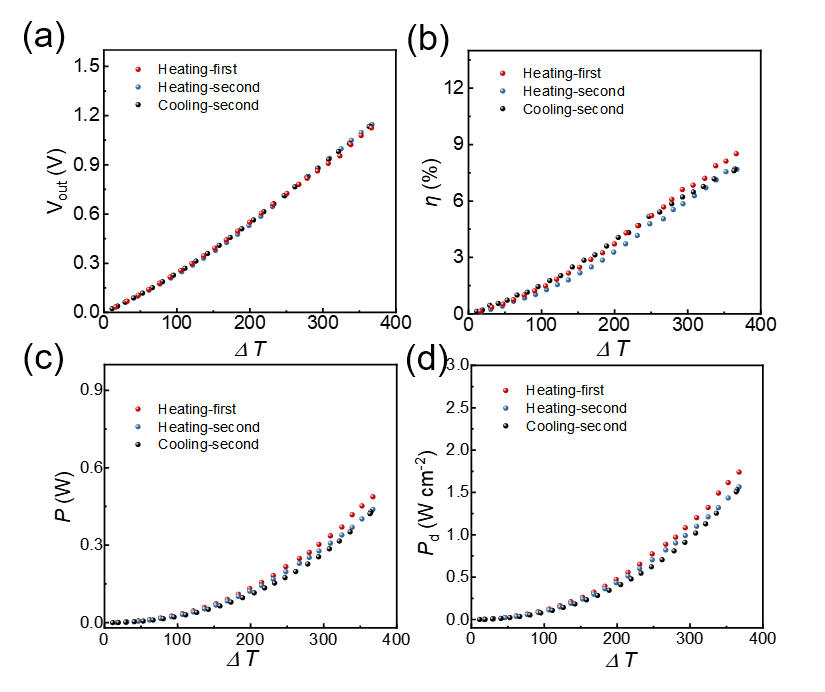


**Figure S22**. Repeatable heating and cooling measurements for 7-pair modular devices. (a) Open-circuit voltage (V_out_), (b) Conversion efficiency (*η*), (c) Output power (*P*), and (d) Output power density (*P*_d_) (with *T*_c_ = 300 K).

**Supplementary Tables**

**Table S1. Room-temperature density of GeTe samples doped with Zr, Cu_2_Te, and Pb.**

| **Sample** | ***ρ* (g/cm^3^)** |
| --- | --- |
| GeTe | 6.14 |
| Ge_0.99_Zr_0.01_Te | 6.11 |
| Ge_0.98_Zr_0.02_Te | 6.09 |
| Ge_0.97_Zr_0.03_Te | 6.08 |
| Ge_0.985_Zr_0.02_Te_0.985_(Cu_2_Te)_0.015_ | 6.06 |
| Ge_0.905_Zr_0.02_Pb_0.06_Te_0.985_(Cu_2_Te)_0.015_ | 6.21 |
| Ge_0.885_Zr_0.02_Pb_0.08_Te_0.985_(Cu_2_Te)_0.015_ | 6.22 |
| Ge_0.865_Zr_0.02_Pb_0.1_Te_0.985_(Cu_2_Te)_0.015_ | 6.27 |
| Ge_0.845_Zr_0.02_Pb_0.12_Te_0.985_(Cu_2_Te)_0.015_ | 6.23 |

**Table S2.** Input parameters used for the theoretical simulation of lattice thermal conductivity in this work.

| **Parameters** | **Values** |
| --- | --- |
| Debye temperature *θ*_D_ (K) | 195 |
| Longitudinal sound velocity *v*_L_ (m/s) | 3442 |
| Transverse sound velocity *v*_T_ (m/s) | 1825 |
| Sound velocity *v*_s_ (m/s) | 2040 |
| $Average atomic mass of \bar{M}$ (kg) | 1.76×10^-25^ |
| $Atomic volume of \bar{V}$ (m^3^) | 2.73×10^-29^ |
| Grain size *d* (m) | 20×10^-6^ |
| Grüneisen parameter *γ* | 1.8 |
| Lattice parameter a (m)  Matrix density *D* (g/cm^3^)  Density difference between the precipitate and matrix Δ*D* (g/cm^3^)  Average radius for the precipitates (nm)  Number density of precipitates *N*p (m^-3^) | 6.03×10^-10^  6.22  1.01  78  4.07×10^16^ |
| Number of stacking faults crossing a line of unit length *N*_s_ (m^-1^) | 1.38×10^6^ |
| Average domain width *d*_BD_ (m) | 68×10^-9^ |
| Point defect scattering parameter Γ | 0.32 (fitted) |
| Domain fitting parameter *A* | 0.074 (fitted) |

**Table S3.** The experimentally measured transport properties of Ge_0.98_Zr_0.02_Te and Ge_0.885_Zr_0.02_Pb_0.08_Te_0.985_(Cu_2_Te)_0.015_ samples.

| **Samples** | **Ge_0.98_Zr_0.02_Te** | **Ge_0.885_Zr_0.02_Pb_0.08_Te_0.985_**  **(Cu_2_Te)_0.015_** |
| --- | --- | --- |
| *n*_H_  (10^20^ cm^-3^) | 4.2 | 0.5 |
| *μ*_H_  (10^20^ cm^-3^) | 45.1 | 59.3 |
| *PF*  (μW cm^-1^ K^-2^) | 8.4 | 19.3 |
| *κ_total_*  (W m^-1^ K^-1^) | 3.81 | 1.38 |
| *zT_max_* | 1.54_(800 K)_ | 2.20_(650 K)_ |

**Supplementary References**

[1] G. Kresse, J. Furthmüller, *Phys. Rev. B* **1996**, *54*, 11169.

[2] J. P. Perdew, K. Burke, M. Ernzerhof, *Phys. Rev. Lett.* **1996**, *77*, 3865.

[3] P. E. Blöchl, *Phys. Rev. B* **1994**, *50*, 17953.

[4] A. van de Walle, M. Asta, G. Ceder, *Calphad* **2002**, *26*, 539.

[5] J. Callaway, H. C. von Baeyer, *Phys. Rev.* **1960**, *120*, 1149.

[6] D. T. Morelli, J. P. Heremans, G. A. Slack, *Phys. Rev. B* **2002**, *66*, 195304.

[7] J. He, S. N. Girard, M. G. Kanatzidis, V. P. Dravid, *Adv. Funct. Mater.* **2010**, *20*, 764.

[8] B. Abeles, *Phys. Rev.* **1963**, *131*, 1906.

[9] B. K. Singh, V. J. Menon, K. C. J. P. R. B. Sood, *Phys. Rev. B* **2006**, *74*, 184302.

[10] D. Wu, L. Xie, X. Xu, J. He, *Adv. Funct. Mater.* **2019**, *29*, 1806613.
